# Transmissible long-term neuroprotective and pro-cognitive effects of 1–42 beta-amyloid with A2T icelandic mutation in an Alzheimer’s disease mouse model

**DOI:** 10.1038/s41380-024-02611-8

**Published:** 2024-06-14

**Authors:** Marina Célestine, Muriel Jacquier-Sarlin, Eve Borel, Fanny Petit, Fabien Lante, Luc Bousset, Anne-Sophie Hérard, Alain Buisson, Marc Dhenain

**Affiliations:** 1 Université Paris-Saclay, CEA, CNRS, Laboratoire des Maladies Neurodégénératives, 18 Route du Panorama, F-92265 Fontenay-aux-Roses, France; 2 Commissariat à l’Energie Atomique et aux Energies Alternatives (CEA), Direction de la Recherche Fondamentale (DRF), Institut de Biologie François Jacob, MIRCen, 18 Route du Panorama, F-92265 Fontenay-aux-Roses, France; 3 Univ. Grenoble Alpes, Inserm, U1216, Grenoble Institut Neurosciences, GIN, 38000 Grenoble, France

**Keywords:** Neuroscience, Physiology

## Abstract

The amyloid cascade hypothesis assumes that the development of Alzheimer’s disease (AD) is driven by a self-perpetuating cycle, in which β-amyloid (Aβ) accumulation leads to Tau pathology and neuronal damages. A particular mutation (A673T) of the amyloid precursor protein (APP) was identified among Icelandic population. It provides a protective effect against Alzheimer- and age-related cognitive decline. This APP mutation leads to the reduced production of Aβ with A2T (position in peptide sequence) change (Aβ_ice_). In addition, Aβ_ice_ has the capacity to form protective heterodimers in association with wild-type Aβ. Despite the emerging interest in Aβ_ice_ during the last decade, the impact of Aβ_ice_ on events associated with the amyloid cascade has never been reported. First, the effects of Aβ_ice_ were evaluated in vitro by electrophysiology on hippocampal slices and by studying synapse morphology in cortical neurons. We showed that Aβ_ice_ protects against endogenous Aβ-mediated synaptotoxicity. Second, as several studies have outlined that a single intracerebral administration of Aβ can worsen Aβ deposition and cognitive functions several months after the inoculation, we evaluated in vivo the long-term effects of a single inoculation of Aβ_ice_ or Aβ-wild-type (Aβ_wt_) in the hippocampus of transgenic mice (APP_swe_/PS1_dE9_) over-expressing Aβ_1-42_ peptide. Interestingly, we found that the single intra-hippocampal inoculation of Aβ_ice_ to mice rescued synaptic density and spatial memory losses four months post-inoculation, compared with Aβ_wt_ inoculation. Although Aβ load was not modulated by Aβ_ice_ infusion, the amount of Tau-positive neuritic plaques was significantly reduced. Finally, a lower phagocytosis by microglia of post-synaptic compounds was detected in Aβ_ice_-inoculated animals, which can partly explain the increased density of synapses in the Aβ_ice_ animals. Thus, a single event as Aβ_ice_ inoculation can improve the fate of AD-associated pathology and phenotype in mice several months after the event. These results open unexpected fields to develop innovative therapeutic strategies against AD.

## Introduction

Alzheimer’s disease (AD) is the most widespread cause of dementia in the world. It is characterized by intracerebral accumulation of abnormal proteinaceous assemblies made of amyloid-β (Aβ) peptides and Tau proteins. Aβ peptides arise from the proteolytic cleavage of the amyloid precursor protein (APP), leading to monomers of Aβ that progressively aggregate to form fibrillary Aβ deposits (Aβ plaques) [1]. The most common forms of Aβ found in the brain are Aβ_1-40_ and Aβ_1-42_ that have 40 and 42 amino acids, respectively. Aβ_1-42_ is more prone to aggregation than Aβ_1-40_ [1]. Aβ plaques can be surrounded by hyperphosphorylated Tau aggregates within neurites and are called neuritic plaques. AD is also characterized by a progressive synaptic dysfunction leading to cognitive deficits [2].

The whole-genome sequencing of the Icelander population revealed an Icelandic mutation (APP_A673T_ or APP_ice_) which protects against AD [3]. The alanine to threonine substitution in the codon 673 of the APP gene occurs within the Aβ sequence close to the β-secretase cleavage site of the APP. The produced Aβ displays A2T (position in peptide sequence) change. A widely advanced hypothesis to explain the protective effect of this mutation is that it shifts APP processing from the amyloidogenic to the non-amyloidogenic pathway leading to a reduced toxic-Aβ production [3–6]. It has also been suggested that Aβ with Icelandic mutation can form Aβ heterodimers with wild-type Aβ [7] and delay aggregation of Aβ peptides [4]. Despite the emerging interest in Aβ with Icelandic mutation during the last decade, their impacts on synaptic impairments and on events associated with the amyloid cascade have never been reported.

Since Aβ is known to induce synaptic deficits [2, 8–11], the first aim of this study was to investigate the impact of Aβ_1-42_ with an A2T Icelandic mutation (herein called Aβ_ice_) on long-term potentiation (LTP) and synaptic health. We showed that Aβ_ice_ protects against Aβ-mediated synaptotoxicity. We then decided to investigate the impact of Aβ_ice_ in vivo in animal models. Several studies in animal models indicate that the intracerebral injections of either Aβ-positive AD brain extracts [12–16] or synthetic or recombinant Aβ peptides [17] induce build-up of Aβ deposits in their host several months after the infusion. Moreover, they can lead to synaptic impairments, long-term cognitive alterations [16, 18–20], and worsen Tau pathology [16]. Here, we wondered if a single inoculation of Aβ_ice_ could provide a long-term protection in an AD mouse model. The effects of Aβ_ice_ were evaluated after a single intra-hippocampal inoculation in transgenic mice over-expressing Aβ_1-42_ peptide and presenting with Aβ plaques and Tau-positive neuritic plaques [16]. Interestingly, this inoculation increased synaptic density, reduced the phagocytosis of synaptic components, rescued spatial memory and lowered Tau-positive neuritic plaques, four months post-inoculation, compared with wild-type Aβ_1-42_ (herein called Aβ_wt_) inoculation. This new unexpected protective action of Aβ_ice_ was not associated with changes of amyloid loads nor with changes of APP processing.

## Methods

Supplementary methods are provided in Supplementary Materials and a key resource table is provided in Supplementary Table 1.

### Primary cultures of cortical neurons: characterisation of dendritic spine density

Mouse cortical neurons were cultured from 14- to 15-day-old OF1 embryos as described previously [21]. Then, different plasmids were generated containing either the WT human APP_695_-mCherry, or the Swedish mutant APP_swe_-mCherry (N595K, L596M) or the Icelandic mutant APP_ice_-mCherry (A598T). Transfections of plasmids containing the APP_x_-mCherry and LifeActin-GFP were performed on cortical neuron cultures after 12 DIV. Then, neurons were visualized using a Nikon Ti C2 confocal microscope with a Nikon 60X water-immersion objective and NIS-Elements software (Nikon, Melville, NY, USA). Following classification rule previously described [22], spines with a minimum head diameter of 0.35 μm and minimum head vs neck ratio of 1.1 were classified as mushroom spines. Non-mushroom spines with minimum volume of 10 voxels (0.040 μm^3^) were classified as stubby spines. All other spines were considered thin (see Supplementary Materials).

### Aβ production measurements *by ELISA assay*

To assess the level of « total » Aβ (secreted into the medium or produced in cell lysate), after 72 h infection of cortical neurons with lentivirus producing various APP_X_ mutants we performed an ELISA assay described in Supplementary Materials.

### Production of recombinant Aβ peptides

Recombinant wild-type human β-amyloid 1–42 protein (Aβ_wt_) and Aβ_ice_ mutant (A2T) were produced as described in Supplementary Materials.

### Electrophysiology recordings

Horizontal brain slices containing the somatosensory cortex were prepared from 20 to 30 day-old OF1 mice. Stimulating electrodes (bipolar microelectrodes) were placed in the stratum radiatum to stimulate the Schaffer collateral pathway. Field EPSPs (fEPSPs) were recorded in the stratum radiatum. For LTP experiments, test stimuli (0.2 ms pulse width) were delivered once every 15 s and the stimulus intensity was set to give baseline fEPSP slopes that were 50% of maximal evoked slopes. Slices that showed maximal fEPSP sizes <1 mV were rejected. Long-term potentiation (LTP) in the hippocampal CA1 region was induced by delivering two 100 Hz protocols (2 × 100 Hz) with an interval of 20 s to the Schaffer collateral/commissural pathway. Aβ peptides were added to the ACSF bath (final concentration of Aβ: 100 nM) 15 min prior to recording. 2 × 100 Hz was delivered after 15 min of stable baseline (see Supplementary Materials).

### Transgenic mice, stereotaxic surgery and behavioral evaluations

In vivo experiments involved the APP_swe_/PS1_dE9_ mouse model of amyloidosis (C57Bl/6 background) [23, 24]. Aβ plaques can be detected as early as 4 months of age in these mice and increase in number and total area with age [23]. This model expresses endogenous murine Tau protein isoforms and is not transgenic for any human Tau. Two-month-old mice received bilateral injections of Aβ_wt_ or Aβ_ice_ solutions (500 µg/mL ( ~ 150 nanomolar) or PBS in the dentate gyrus. The dose used here is in the same range as doses previously published [13] − 5 ng/µl - Bilateral injections of 2.5 μl of Aβ; [25] − 250 µg/ml with 30 µl inoculated). Animals were studied 4 months post-inoculation (mpi). Group sizes were, respectively, *n*_*APP/PS1-A*βice_ = 12, *n*_*APP/PS1-A*βwt_ = 11, *n*_*APP/PS1-pbs*_ = 10. Wild-type littermates injected with the PBS were used as controls for the behavioral tests (*n*_*WT-pbs*_ = 10). Females were exclusively used in this study in order to optimize group homogeneity (Aβ plaque load is known to vary between males and females). Spatial memory was evaluated using the Morris water maze. All experimental procedures were conducted in accordance with the European Community Council Directive 2010/63/UE and approved by local ethics committees (CEtEA-CEA DSV IdF N°44, France) and the French Ministry of Education and Research (A20_017 authorization), and in compliance with the 3 R guidelines. Further information on the animal experiments are provided in Supplementary Materials.

### Mouse brain analysis

Mice were euthanized at 4 mpi, after the behavioral tests. Their left hemisphere was dissected in order to take out the hippocampus and the cortex for biochemistry analysis performed based on standard protocols of western blot and dot blot analysis. The right hemisphere was used for histology. Aβ deposits were examined using a 4G8 (Biolegend 800706) labeling. Tau was examined using labeling with AT8 (Thermo MN1020B) directed against hyperphosphorylated Tau. Astrocytes were stained with the GFAP antibody (Dako Z0334). Microglia were evaluated using Iba1 (Wako 1919741), commonly considered a pan-microglial marker and associated with motility [26] as well as CD68, a lysosome marker (Biorad MCA1957). Synaptic density was assessed in the hippocampus (CA1) and the perirhinal/entorhinal cortex using a double immunolabeling of presynaptic (Bassoon) and postsynaptic (Homer1) markers as previously reported by our group [16]. The colocalisation between CD68+ microglial lysosomes and postsynaptic Homer1 marker was performed using Imaris [27]. Further information on the protocols are available in Supplementary Materials.

### Aβ deposits and Tau-positive neuritic plaques analysis

Stained sections were scanned using an Axio Scan.Z1. Image processing and analysis were performed with ImageJ. 4G8-positive amyloid and AT8-positive tau loads were evaluated through automatic local threshold using the Phansalkar method (radius = 15). In APP_swe_/PS1_dE9_ mice, tau lesions occur in the form of neuritic plaques *i.e*. tau aggregates within neurites surrounding Aβ deposits. The AT8-positive area presenting within neuritic plaques was evaluated by drawing circular regions of interest (with a constant area of 6 µm²), and by quantifying the percentage of tau-positive regions within each ROI, using the thresholding method previously described (see Supplementary Materials).

### Statistical analysis

A detailed description of the statistical analyses is provided in Supplementary Materials.

## Results

### APP with the Icelandic mutation is not synaptotoxic when expressed in cortical neurons

**Fig. 1:**
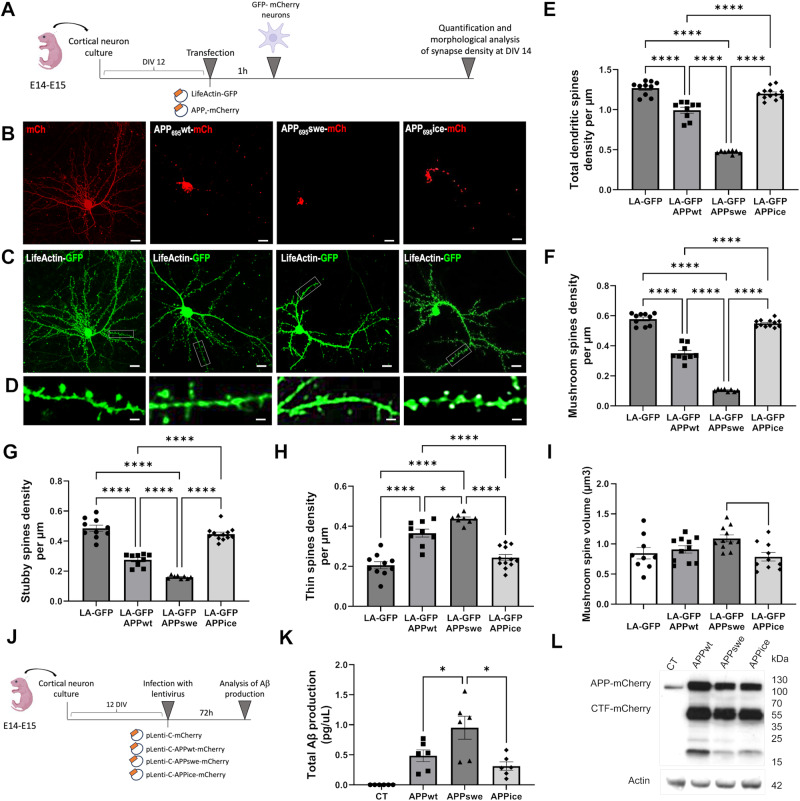
Over-expression of APP_ice_ in cortical cell cultures protects spine density but does not influence Aβ production. **A** Schematic illustration of primary cortical neuron transfection and analysis. Neuronal cells were isolated from the neocortices of 14- to 15-day embryos. Transfection with APP_x_-mCherry and LifeActin-GFP were performed after 12 DIV. Plasmid were applied to cells for 60 min. Finally, neurons were visualized and their morphology and density were analyzed according to a classification rule [22]. Representative images of cultured cortical neurons over-expressing mCh (control neurons), APP_wt_-mCh, APP_swe_-mCh, APP_ice_-mCh (**B**) and LifeActin-GFP (LA-GFP) (scale bar = 10 µm) (**C**). **D** Representative dendrite portions (scale bar = 5 µm). **E** Reduction of total spine density in APP_wt_ (****p < 0.0001 compared with control neurons and APP_ice_) and APP_swe_ (****p < 0.0001 compared with control neurons, APP_wt_ and APP_ice_) while APP_ice_ does not modulate total spine density (p = 0.5 compared with control neurons). **F** Reduction of mushroom spine density in APP_wt_ (****p < 0.0001 compared with control neurons and APP_ice_) and in APP_swe_ (****p < 0.0001 compared with control neurons, APP_wt_ and APP_ice_) while APP_ice_ does not modulate mushroom spine density (p = 0.6 compared with control neurons). **G** Reduction of stubby spine density in APP_wt_ (****p < 0.0001 compared with control neurons and APP_ice_) and in APP_swe_ (****p < 0.0001 compared with control neurons, APP_wt_ and APP_ice_). **H** Increase of thin spine density in APP_wt_ (****p < 0.0001 compared with control neurons and APP_ice_) and in APP_swe_ (****p < 0.0001 compared with control neurons and APP_ice_; *p = 0.012 compared with APP_wt_). **I** Increase of mushroom spine volume in APP_wt_ (*p < 0.05 compared with control neurons and APP_ice_) and in APP_swe_ (**p < 0.01 compared with control neurons and APP_ice_; *p = 0.012 compared with APP_wt_), while APP_ice_ does not modulate mushroom spine volume (p = 0.6 compared with control neurons). nLA-GFP = 10, nAPP_wt_ = 9, nAPP_swe_ = 8, nAPP_ice_ = 12 neurons from at least 3 different cultures. **J** Schematic illustration of neural infections by viral vectors **K** Quantification of total Aβ productions from neurons infected with APP_wt_, APP_swe_ or APP_ice_. Cortical neurons infected with APP_swe_ produce an increased level of Aβ compared with APP_wt_ (*p = 0.045) and APP_ice_ (*p = 0.010) while production of Aβ is similar between APP_wt_ and APP_ice_ (p = 0.36 in ANOVA group comparison; p = 0.309 in Mann–Whitney test). **L** Western blot of infected neuron lysate showing no difference in overall APP levels using Y188 antibody. N = 6 different cortical neuron cultures. *p < 0.05, ****p < 0.0001. Data are shown as mean ± s.e.m.

Several studies have reported that the Icelandic mutation (APP_ice_) is protective because it reduces Aβ production. To further investigate its biology, we over-expressed mutated human neuronal forms of APP_ice_ in primary mouse cortical neurons. First, we wondered whether APP_ice_ had specific effects on spine density and morphology of dendritic synapses. To do so, we selected two forms of human APP: a non-mutated form (APP_wt_) and APP with Swedish mutation (K595N/M596L, APP_swe_). Both were compared with APP_ice_. All APP variants were fused to mCherry (mCh) and cortical neurons were co-transfected with LifeActin-GFP (LA-GFP), a small peptide that specifically binds to filamentous actin without disrupting actin stoichiometry (Fig. 1A-C). This latter enables the visualization of the dendritic arbor and spines (Fig. 1D). Neurons over-expressing APP_wt_- and APP_swe_-mCh displayed a marked and significant decrease in total spine density compared with control neurons that over-expressed mCherry and LA-GFP (Fig. 1E). On the contrary, over-expression of APP_ice_-mCh had no effect on total spine density (Fig. 1E). Then, we looked at spine subpopulations: mature (mushroom, stubby) and immature (thin) spines. We found a highly significant decrease of mushroom (Fig. 1F) and stubby (Fig. 1G) spine density when APP_wt_- or APP_swe_-mCh were over-expressed compared with control (Fig. 1F-G). There was also a significant increase in thin spine density for neurons over-expressing APP_wt_- and APP_swe_-mCh (Fig. 1H). APP_ice_-mCh had no effect on mushroom, stubby or thin spine density and distribution (Fig. 1F-H). Moreover, increased volumes of mushroom spines were found in APP_wt_- and APP_swe_-mCh expressing neurons compared with control while APP_ice_-mCh had no effect (Fig. 1I). Altogether, these results show that, unlike APP_wt_, APP with icelandic mutation is non-synaptotoxic even after its over-expression in neurons.

To decipher whether the effects observed in APP-transfected neurons were due to changes in Aβ production or/and an effect induced by the mutated APPs alone, we evaluated the amount of Aβ in neurons expressing APP_wt_, APP_swe_ or APP_ice_ (Fig. 1J, K). APP_swe_ increased Aβ production compared to APP_wt_ (46.6%, p = 0.045) while for APP_ice_, Aβ production was significantly reduced compared to APP_swe_ (64%, p = 0.01). APP_ice_ reduction compared to APP_wt_ did not reach the significant level (17.3%, p = 0.36) (Fig. 1K). Furthermore, neurons over-expressed similar levels of APP that was processed into C-terminal fragments (CTF) (Fig. 1L).

### Aβ_1-42_ with A2T Icelandic mutation (Aβ_ice_) is not synaptotoxic

**Fig. 2:**
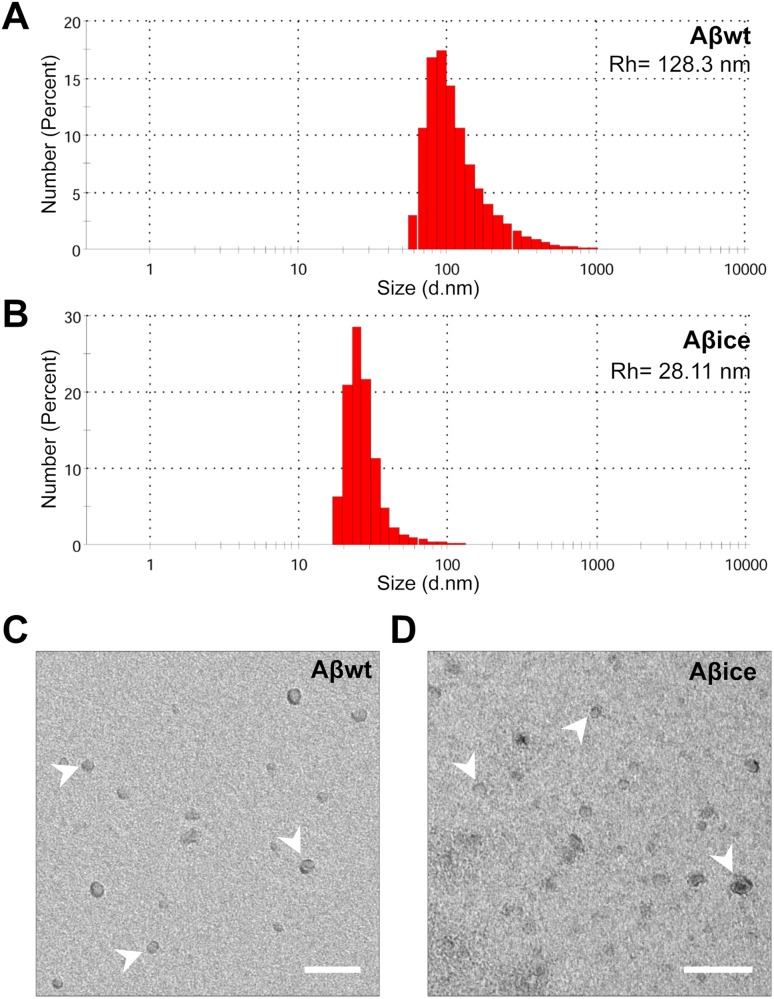
Characterization of Aβ_wt_ and Aβ_ice_ samples. **A**, **B** Particle size analysis by dynamic light scattering to assess hydrodynamic radius (Rh). Size distribution by number of particles (%) at 30 µM had peak averages Rh of 128.3 nm for Aβ_wt_ (**A**) and 28.11 nm for Aβ_ice_ (**B**). Representative electron microscopy images of Aβ assemblies (arrows) in Aβ_wt_ (**C**) and Aβ_ice_ (**D**) solution showing lack of fibrillary assemblies. Scale bars: 100 nm.

**Fig. 3:**
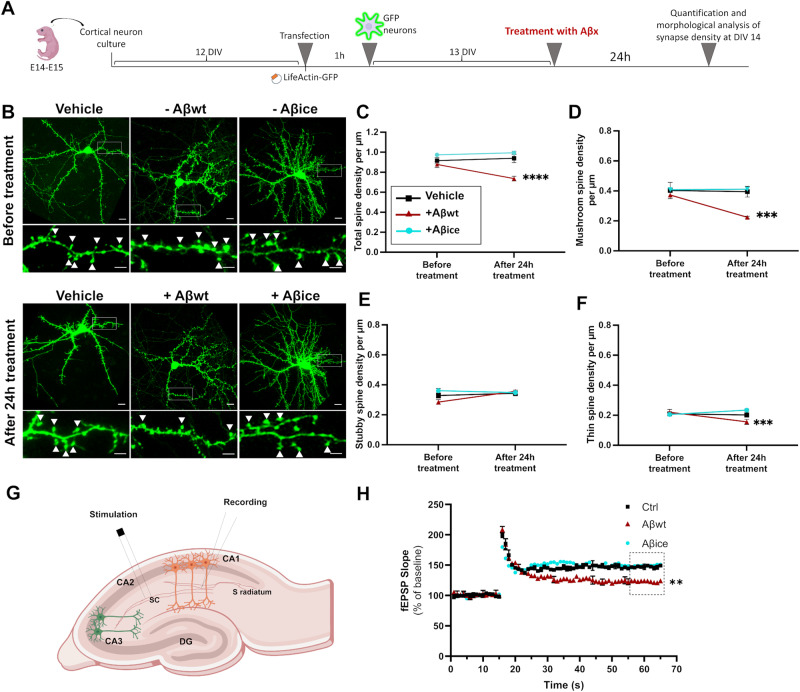
Exogenous application of Aβ_ice_ is not synaptotoxic. **A** Schematic illustration of primary cortical neuron transfection and analysis. Neuronal cells were isolated from the neocortices of 14- to 15-day embryos. Transfections with LifeActin-GFP plasmid were performed after 12 DIV. Plasmid were applied to cells for 60 mins. Neurons were seeded and maintained at 37 °C. Treatments with 100 nM of Aβ_ice_ or Aβ_wt_ were performed on neuronal cultures at 13 DIV during 24 h. Finally, neurons were visualized at DIV 14 and their morphology and density were analyzed according to a classification rule [22]. **B** Representative images of primary cultures of cortical neurons expressing LA-GFP before (top), and after (bottom) treatment for 24 h with Aβ_ice_ or Aβ_wt_. Top row wide field view, scale bar = 10 µm; bottom row: dendrite portions with mushroom spines (white arrows, scale bar = 5 µm). **C** Quantification of total spine density showed a reduction of total number of spines after treatment with Aβ_wt_, compared to PBS (****p < 0.0001) and Aβ_ice_ (****p < 0.0001) while no difference was observed after treatment with Aβ_ice_ compared to PBS (p = 0.46). **D** Quantification of mushroom spine density showed a reduction of the number of mushroom spines after treatment with Aβ_wt_ compared to PBS and Aβ_ice_ (***p = 0.0004 and p = 0.0001, respectively) while no difference was observed after treatment with Aβ_ice_ compared to PBS (p = 0.91). **E** Stubby spine density was not modified after treatment with the different Aβ seeds. **F** Quantification of thin spine density showed a reduction of the number of thin spines after treatment with Aβ_wt_ compared to Aβ_ice_ (***p = 0.0005). n = 6 neurons from at least 3 different cultures. Data are shown as mean ± s.e.m. Kruskal-Wallis with Dunn’s multiple comparisons. **G** Long-term potentiation (LTP) in the hippocampal CA1 region was induced by delivering stimulations to the Schaffer collateral/commissural (sc) pathway. Aβ_wt_ or Aβ_ice_ peptides were added to the artificial CSF bath 15 min prior to recording. **H** Stimulations were delivered after 15 min of stable baseline. Each point on the graph represents the mean ± s.e.m. While Aβ_ice_ did not modulate LTP (p > 0.9), Aβ_wt_ decreased LTP (p = 0.0017) when comparing the last 10 time points of fEPSP slope (% of baseline) to control conditions (Ctrl; without Aβ treatment). n = at least 5 slices per condition. **p < 0.01, *** p < 0.001, **** p < 0.0001.

We then decided to focus on Aβ_1-42_ with A2T icelandic mutation (Aβ_ice_) and not on APP_ice_. The 1-42 form was chosen as Aβ_1-42_ is the most toxic form of Aβ found in AD brains. Thus, we produced Aβ_1-42_ with A2T icelandic mutation (Aβ_ice_) and compared its synaptotoxicity with that of a wild-type form of Aβ_1-42_ (Aβ_wt_). Aβ_wt_ and Aβ_ice_ oligomeric species were characterized by dynamic light scattering and electron microscopy. The hydrodynamic radius of monomeric Aβ_1-42_ usually ranges from 0.9 to 1.4 nm [28]. Aβ_wt_ and Aβ_ice_ display high hydrodynamic radius of 128.3 and 28.11 nm respectively (Fig. 2A-B), indicative of a high degree of oligomerization. Although dynamic light scattering does not allow precise estimation of the number of molecules within oligomers, Aβ_wt_ particles appeared larger than Aβ_ice_. The electron micrographs showed spheroid particles without detectable fibrillar assemblies (Fig. 2C, D). The acute toxicity of Aβ_ice_ and Aβ_wt_ seeds on synaptic health were then evaluated by assessing spine morphology of primary cortical neuron cultures. Cortical neurons were co-transfected with LifeActin-GFP (LA-GFP) and then incubated with 100 nM of either Aβ_ice_ or Aβ_wt_ for 24 h (Fig. 3A). We quantified thin, stubby, and mushroom spine density before and after Aβ peptides treatment (Fig. 3A–F). A spine loss was observed in Aβ_wt_-treated neurons compared to vehicle (p < 0.05, Fig. 3C). The spine loss was mainly due to a reduction in mushroom (p < 0.005, Fig. 3D) and thin spine (p < 0.005_,_ Fig. 3F) and not stubby spine (Fig. 3E) densities. On the contrary, the total spine density (Fig. 3C) of neurons treated with Aβ_ice_ as well as their sub-population (Fig. 3D–F) was similar to the vehicle condition, which suggests that Aβ_ice_ was not synaptotoxic. We then evaluated whether Aβ_ice_ can modulate positively hippocampal synaptic activity, basal synaptic transmission, short and long-term plasticity in wild-type mouse brain slices after exogenous application of Aβ_ice_ or Aβ_wt_. Both peptides were pre-incubated with slices for 15 min before the experiments. Field recording of postsynaptic excitatory (fEPSP) responses was elicited by CA3-CA1 collateral fibers electrical stimulation (Fig. 3G). The efficacy of basal synaptic transmission was determined by a range of electrical stimuli from 10 to 100 μA (Supplementary Fig. 1A) and paired-stimulation with an inter-pulse interval from 25 to 300 ms was performed to evaluate short-term plasticity (Supplementary Fig. 1B). Both input/output (I/0) curves and paired pulse facilitation ratio (PPR) did not reveal any significant differences between all conditions. In contrast, one hour after 100 Hz electric stimulation induced hippocampal long-term potentiation (LTP) LTP, we observed an increase in the average fEPSP slope in PBS (control) and Aβ_ice_–exposed slices, compared with a moderate increase in Aβ_wt_ exposed slices. Thus, Aβ loses its deleterious effect on long-term plasticity when it bears the Icelandic mutation, since Aβ_wt_ acutely impaired LTP in vitro, while Aβ_ice_ did not (Fig. 3H).

### Aβ_1-42_ with A2T Icelandic mutation (Aβ_ice_) rescues APP-associated synaptic toxicity in vitro

**Fig. 4:**
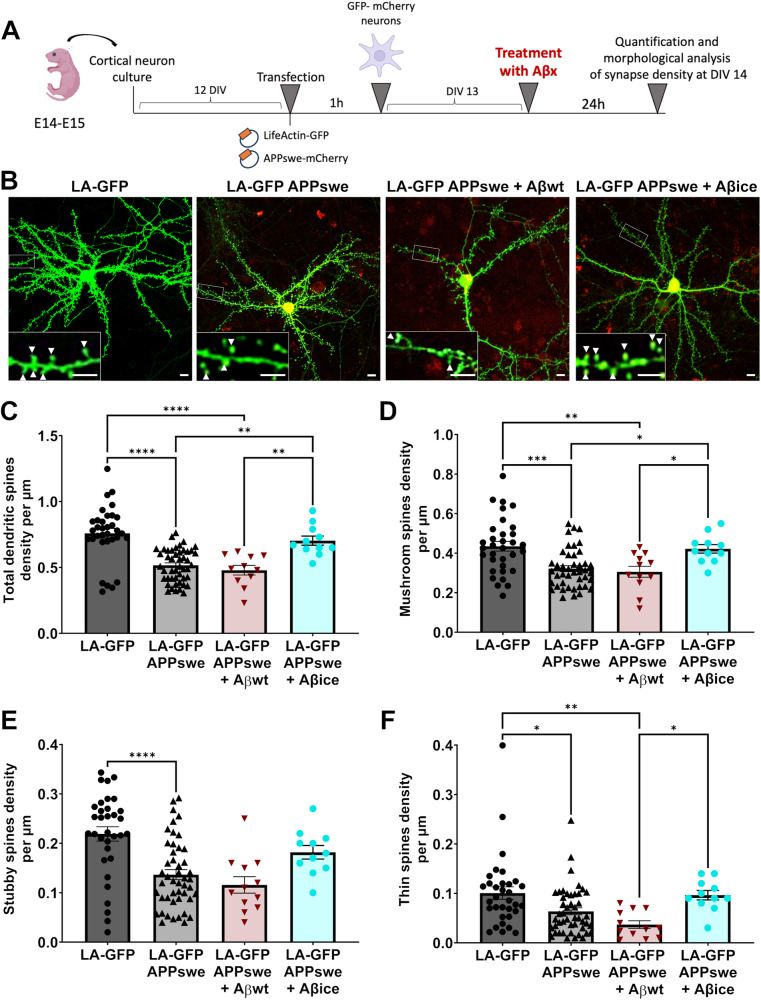
Exogenous application of Aβ_ice_ protects against Aβ-mediated synaptic alterations. **A** Schematic illustration of primary cortical neuron transfection and analysis. Neuronal cells were isolated from the neocortices of 14- to 15-day embryos. Transfection with APP_x_-mCherry and LifeActin-GFP plasmid were performed after 12 DIV. Plasmid were applied to cells for 60 mins. Neurons were seeded and maintained at 37 °C. Treatments with 100 nM of Aβ_ice_ or Aβ_wt_ were performed on neuronal cultures at 13 DIV during 24 h. Finally, neurons were visualized at DIV 14 and their morphology and density were analyzed according to a classification rule [22]. **B** Representative images of cultured cortical neurons expressing LA-GFP (left), expressing LA-GFP and APP_swe_ after a 24 h incubation with buffer (middle) or Aβ_ice_ (right). Scale bar = 10 µm. Inset: Dendrite portions presenting with less mushroom spines (white arrows) in APP_swe_ neurons but not after 24 h incubation with Aβ_ice_. Scale bar = 5 µm. **C** Reduction of total spine density in APP_swe_ and after Aβ_wt_ treatment (****p < 0.0001 compared with control LA-GFP alone) while recovery after treatment with Aβ_ice_ was observed (**p = 0.002 compared with APP_swe_; **p = 0.003 compared with APP_swe_ + Aβ_wt_). **D** Reduction of mushroom spine density in APP_swe_ (***p = 0.0002 compared with control LA-GFP alone) and after Aβ_wt_ treatment (**p = 0.004 compared with control LA-GFP alone) while a recovery of mushroom spine density was observed after treatment with Aβ_ice_ (*p = 0.036 compared with APP_swe_; **p = 0.042 compared with APP_swe_ + Aβ_wt_). Quantification of **E** stubby spine and **F** thin spine density showing a reduction of stubby and thin spine number in APP_swe_ (**E** ****p < 0.0001; **F** *p = 0.017 compared with control LA-GFP) and after Aβ_wt_ treatment (**F** **p = 0.0045 compared with control LA-GFP; *p = 0.039 compared with APP_swe_ + Aβ_ice_). Quantification of both do not change after treatment with Aβ_ice_ (**D** p = 0.116 ; **E** p = 0.211 compared with APP_swe_). nLA-GFP = 33, nAPP_swe_ = 47, nAPP_swe_ + Aβ_WT_ = 12; nAPP_swe_ + Aβ_ice_ = 11 neurons n = 6 neurons from at least 3 different cultures. *p < 0.05, **p < 0.01, ***p < 0.001 ****p < 0.0001. Data are shown as mean ± s.e.m.

Then, we wondered if Aβ_ice_ modulates positively synapse morphology and density impaired by APP/Aβ-related toxicity. To model in vitro Aβ-associated AD-like pathology, we used primary cortical neurons that over-express APP transgene bearing the Swedish mutation (APP_swe_) leading to an overproduction of endogenous non-mutated forms of Aβ. We compared spine morphology of APP_swe_ expressing cortical neurons alone and after 24 h of Aβ_wt_ or Aβ_ice_ exposition. As a control, we also imaged LifeActin-GFP (LA-GFP) cortical neurons that do not express the transgene (Fig. 4A, B). We analyzed total dendritic spine density as well as mushroom, stubby and thin spine density (Fig. 4C–F). While APP_swe_ expressing neurons exhibited a significant decrease in spine density, the exogenous application of Aβ_ice_ (but not of Aβ_wt_) led to an increase in the total spine density and canceled this significant decrease (Fig. 4C). This spine loss induced by APP_swe_ expression involved mushroom, stubby and thin spines (Fig. 4D–F). Treatment by Aβ_ice_, but not by Aβ_wt_, mainly restored mushroom spine density (Fig. 4D). These results indicate that a 24 h exposition to Aβ_ice_ rescues the synaptotoxicity induced by APP_swe_ expression in neurons.

### A single inoculation of Aβ_ice_ promotes spatial memory and synaptic density in mice

**Fig. 5:**
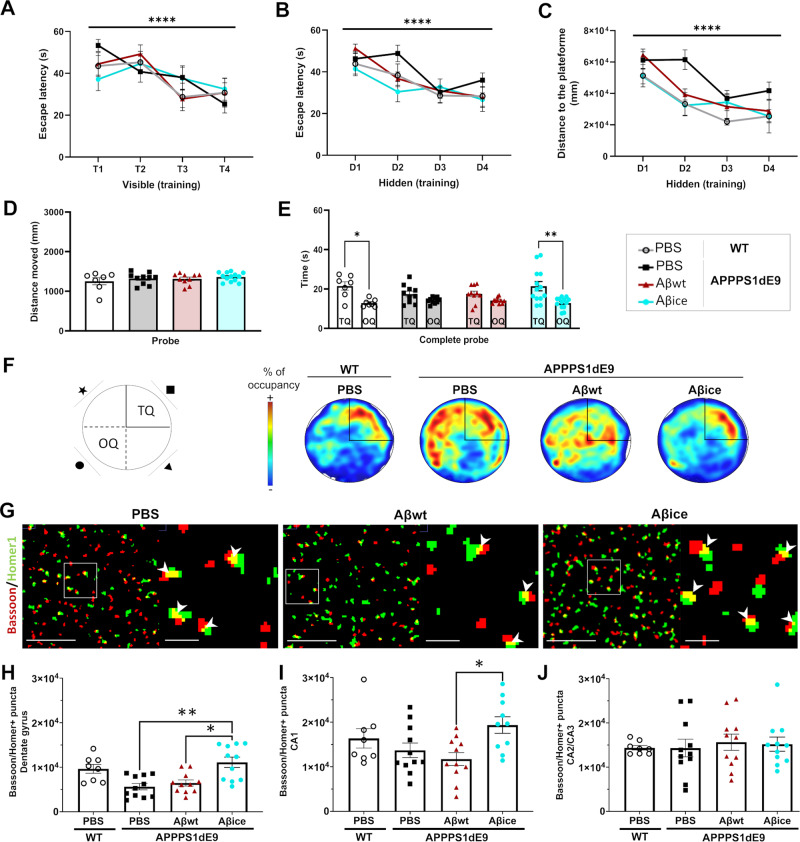
Aβ_ice_ intrahippocampal infusion rescues spatial memory and increases synaptic density. Spatial memory was evaluated using Morris water maze tasks at 4 months post-inoculation. **A** During the visible platform phase, escape latencies decreased across the four trials (F_(2.950, 177.0)_ = 11.76, p < 0.0001). No difference was observed between the groups (F_(4, 63)_ = 0.07, p > 0.9). WT mice and APP_swe_/PS1_dE9_ mice inoculated with PBS, Aβ_wt_ or Aβ_ice_ had comparable learning abilities, as suggested by the decrease in (**B**) time to find the platform (for the days: F_(1.69, 82.98)_ = 18.65; p < 0.0005) and (**C**) the distance moved (for the days: F_(2.894, 101.3)_ = 20.74, p < 0.0005) throughout the 4 training day. **D** The distance moved during the probe test was similar between groups. **E** During the probe test evaluating spatial memory, the time spent in the target quadrant (TQ) was significantly higher than the time spent in the opposite one (OQ) in WT mice (p = 0.038) and Aβ_ice_-inoculated APP_swe_/PS1_dE9_ mice (p = 0.003). **F** Representative heatmap images of probe test. Increased red color intensity represents increased time spent in a given location. Conversely, a cooler color indicates a shorter time spent in the location. The TQ is represented. Unlike WT animals, PBS and Aβ_wt_-inoculated APP_swe_/PS1_dE9_ mice were unable to find the target platform. Aβ_ice_ rescued this phenotype. **G** Representative views of original Bassoon/Homer segmented and co-localized puncta (white arrow). Scale bars: main images: 5 µm; Insets: 1 µm (**H**–**J**). In the dentate gyrus (**H**), a decrease of co-localized puncta between Bassoon and Homer synaptic markers is observed in APP_swe_/PS1_dE9_ animals treated with PBS (13.9%) or Aβ_wt_ compared to WT animals. An increase of co-localized puncta between Bassoon and Homer synaptic markers is observed in Aβ_ice_-inoculated mice in (**H**) the dentate gyrus (**H**: PBS- versus Aβ_ice_-inoculated APP_swe_/PS1_dE9_: p = 0.005 and Aβ_wt_- versus Aβ_ice_-inoculated APP_swe_/PS1_dE9_: p = 0.03) and (**I**) the CA1 (**I**:Aβ_wt_- versus Aβ_ice_-inoculated APP_swe_/PS1_dE9_: p = 0.01). Synaptic density in the CA2/3 (**J**) was similar between groups (p = 0.86). nWT_PBS_ = 7, nAPP/PS1_PBS_ = 10, n_Aβwt_ = 11, n_Aβice_ = 12 mice.*p < 0.05, **p < 0.01, ***p < 0.001 ****p < 0.0001. Data are shown as mean ± s.e.m.

We next wanted to assess whether Aβ_ice_-related beneficial effects could be obtained in AD mice which highly express the human form of APP transgene bearing the Swedish mutation. Aβ_ice_ or Aβ_wt_ were inoculated in the dentate gyrus of the hippocampus of APP_swe_/PS1_dE9_ mice at the age of 2 months. An additional group of PBS-inoculated wild-type (WT) littermates was used as control. Behavioral assessment of mice was performed 4 months post-inoculation (mpi). Spatial memory was evaluated using the Morris water maze. We first ensured that mice could swim and did not have visual deficiency using a visible platform (Fig. 5A). Then, 4-days training phase allowed mice to learn the location of the hidden platform (training phase). The day of the probe test, we removed the platform to assess mouse recall and we recorded the time spent in the quadrant of the platform (target quadrant) versus the other quadrants. Mice from each group showed similar spatial learning as suggested by comparable reduction of the time spent to find the hidden platform over the training days (Fig. 5B). Spatial memory was evaluated 24 h after training. Interestingly, Aβ_ice_-inoculated APP_swe_/PS1_dE9_ and WT mice significantly spent more time in the target quadrant than the other quadrants compared with PBS- or Aβ_wt_-inoculated APP_swe_/PS1_dE9_ mice. This suggests that spatial memory is impaired in 6-month-old APP_swe_/PS1_dE9_ mice and that a single inoculation of Aβ_ice_ rescues spatial memory four months following the inoculation (Fig. 5E, F).

Mice were euthanized 4 mpi to evaluate cerebral lesions. First, we investigated synaptic density at the inoculation site (hippocampus). Double immunolabeling of presynaptic (Bassoon) and postsynaptic (Homer) markers was performed and the amount of colocalized puncta was quantified as an indicator of synaptic integrity (Fig. 5G). Synaptic density was reduced in APP_swe_/PS1_dE9_ mice inoculated with PBS or with Aβ_wt_ compared to wild-type animals (Fig. 5H). Interestingly, Aβ_ice_-inoculated APP_swe_/PS1_dE9_ mice had increased synaptic densities in the dentate gyrus and CA1 compared with mice inoculated with PBS or Aβ_wt_, and their synaptic densities were similar to those of wild-type animals (Fig. 5H–I). Synapses in the CA2/3 region were not modulated **(**Fig. 5J**)**. Thus, Aβ_ice_ has a synapto-protective effect in vivo after a single intra-hippocampal inoculation. This effect is associated with increased spatial memory.

### Aβ_ice_ inoculation does not change amyloid plaque load in vivo

**Fig. 6:**
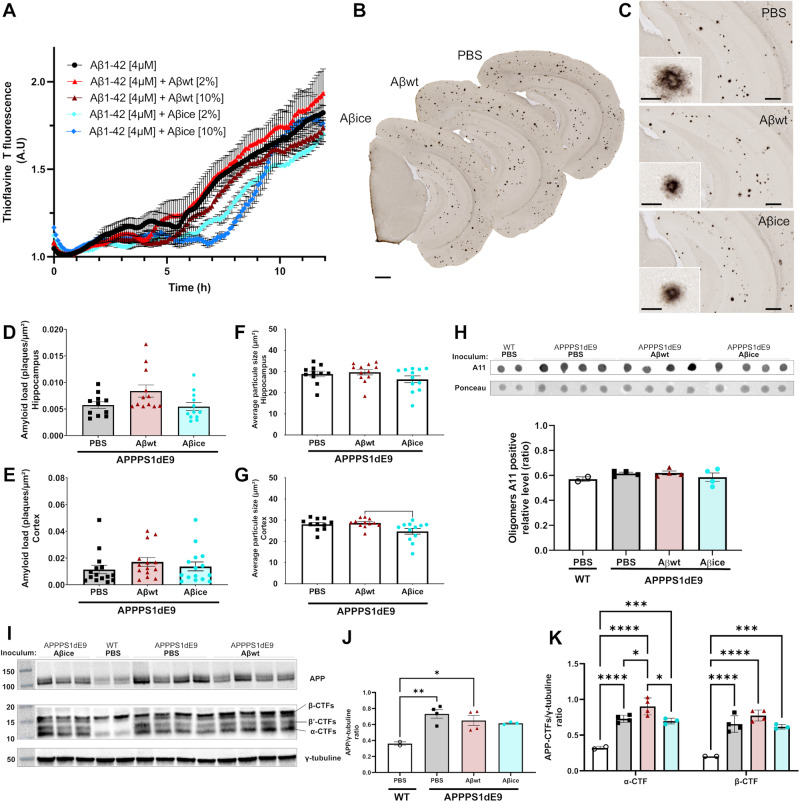
Aβ aggregation, Aβ deposition and APP processing profiles at 4 mpi. **A** Kinetics of synthetic Aβ_1-42_ aggregation monitored by Thioflavin T (ThT) fluorescence in the absence and presence of Aβ_wt_ and Aβ_ice_ seeds (data issued from three independent kinetic experiments). Aggregation experiments were performed in triplicate. The aggregation curves were normalized to maximal values of ThT fluorescence at plateau. An elongation of the lag time was observed by seeding with Aβ_ice_ [10%] compared to Aβ_1-42_ alone (p = 0.035, Kruskal-Wallis test). **B** Representative images of 4G8 immunolabeling showing Aβ plaque deposition in the brain of APP_swe_/PS1_dE9_ mice after PBS, Aβ_wt_ or Aβ_ice_ inoculation in the dentate gyrus. **C** Representative images of Aβ plaque deposition in the hippocampus. Magnified views showed no difference in the morphology of Aβ plaques between groups. **D**, **E** Quantification of amyloid load (4G8-positive Aβ plaques per µm²) revealed no difference between groups in the hippocampus (**D** p = 0.09) and in the cortex (**E** p = 0.2). nAPP/PS1_PBS_ = 10, n_Aβwt_ = 11, n_Aβice_ = 12 mice. Average amyloid plaque size in the hippocampus (**F**) and cortex (**G**). A reduction in the average amyloid plaque size is observed in the cortex of Aβ_ice_-inoculated APP_swe_/PS1_dE9_ mice compared to Aβ_wt_-inoculated APP_swe_/PS1_dE9_ (p = 0.028). **H** Dot blot analysis for oligomeric species (A11) in sarkosyl-soluble extract from the hippocampus of APP_swe_/PS1_dE9_ mice after PBS, Aβ_wt_ or Aβ_ice_ inoculation at 4mpi. Similar relative expression levels of A11 are observed between groups. **I** Western-blot analysis (APP-Cter-17 antibody [52]) of total endogenous APP, APP-CTFs and tubulin in hippocampus lysates (S100K fractions) obtained from wild-type and APP_swe_/PS1_dE9_ mice after PBS, Aβ_wt_ or Aβ_ice_ inoculation. Full length APP runs at an apparent molecular size of 110 kDa, β-, β′- and α-CTF are detected at 16 kDa, 12 kDa and 11 kDa respectively. Tubulin staining was used as a marker and loading control. **J** The semi-quantification of total APP reveals similar levels of APP in PBS-, Aβ_wt_- or Aβ_ice_-inoculated APP_swe_/PS1_dE9_ mice. APP levels were significantly higher in PBS- (**p = 0.007) and Aβ_wt_- (*p = 0.029) inoculated APP_swe_/PS1_dE9_ mice than WT mice. **K** Semi-quantification of β-CTF/C99 and α-CTF/C83. An increase level of both fragments were observed in PBS- (****p < 0.0001), Aβ_wt_- (****p < 0.0001) or Aβ_ice_- (***p = 0.0001) inoculated APP_swe_/PS1_dE9_ mice compared with PBS-inoculated WT mice. Similar level of CTFs were shown between PBS- and Aβ_ice_-inoculated APP_swe_/PS1_dE9_ mice (p = 0.99). An increase level of α-CTF was observed in Aβ_wt_-inoculated APP_swe_/PS1_dE9_ mice compared with PBS- (*p = 0.035) and Aβ_ice_- (*p = 0.018) inoculated APP_swe_/PS1_dE9_ mice. nWT_PBS_ = 2, nAPP/PS1_PBS_ = 4, nAPP/PS1_Aβwt_ = 4, nAPP/PS1_Aβice_ = 3 mice. *p < 0.05, **p < 0.01, ***p < 0.001, ****p < 0.0001. Data are shown as mean ± s.e.m. Scale bars: B: 500 µm; **C** main images = 100 µm, Insets = 20 µm.

Aβ aggregation is a nucleation-dependent polymerization process, with a slow initial nucleation phase, called lag-phase, followed by a rapid growth phase [29]. We investigated the seeding properties of both Aβ peptides in vitro using thioflavin fluorescence assay [30, 31]. First, synthetic monomeric Aβ_1-42_ was incubated at 37 °C, the ThT fluorescence signal displayed a sigmoidal shape characterized by a 6 h lag time followed by an 8 h elongation step (Fig. 6A). When synthetic monomeric Aβ_1-42_ was seeded with recombinant Aβ_wt_ [2 and 10%, v/v], assembly kinetics was not affected while Aβ fibrils were formed (Fig. 6A). On the contrary, addition of Aβ_ice_ [2 and 10%, v/v] delayed the lag time to 1 and 2 h respectively (Fig. 6A). Thus, Aβ_ice_ hindered Aβ aggregation.

To move one step forward, we investigated the impact of in vivo Aβ inoculation on amyloid load. In a previous in vivo study we showed that inoculation of toxic forms of Aβ (with an Osaka mutation) increases amyloid load, Aβ oligomers and modulates APP processing in mice [20]. Here, we wondered whether the inoculation of Aβ_ice_ could have the opposite effects. First, we observed Aβ deposits in the hippocampus and the cortex of all inoculated APP_swe_/PS1_dE9_ mice at 4 mpi (Fig. 6B, C). Aβ plaque loads were similar between groups in the hippocampus (Fig. 6D) and the cortex (Fig. 6E). However, amyloid plaque sizes were significantly lower in the cortex of Aβ_ice_-inoculated animals compared with Aβ_wt_-inoculated animals (Fig. 6G). This effect was not detected in the hippocampus (Fig. 6F). Then, we fractionated soluble and insoluble Aβ aggregates from the hippocampus by sarkosyl detergent extraction. Dot blot analysis of sarkosyl-soluble fraction using conformational antibody against oligomers (A11) did not show any change in Aβ oligomer profiles in Aβ_ice_- or Aβ_wt_-inoculated animals as compared with PBS (Fig. 6H).

The amyloidogenic processing accounts for only 10% of total APP processing. The remaining processing serves to generate different N- or C-terminal fragments of APP that are thought to have a crucial role in AD pathophysiology. We further evaluated APP proteolytic profiles caused by α- or β-secretase pathways in the hippocampus at 4 mpi. We showed that all inoculated APP_swe_/PS1_dE9_ mice produced similar levels of human APP, as confirmed by western blot analysis (Fig. 6I, J), that is processed into several C-terminal fragments (CTF): α-CTF (C83, 9kDA), β-CTF (C99, 11 kDa) and β’-CTF (15 kDa). Overall, PBS, Aβ_ice_- or Aβ_wt_-inoculated animals produced more CTF in their hippocampus than WT mice (Fig. 6K). Aβ_wt_-inoculated APP_swe_/PS1_dE9_ mice had increased APP processing as suggested by a higher level of α-CTF products compared with PBS and Aβ_ice_-inoculated APP_swe_/PS1_dE9_ mice (Fig. 6K). Interestingly, we did not find any change in CTF hippocampic levels following Aβ_ice_ inoculation compared with PBS-inoculation in APP_swe_/PS1_dE9_ mice (Fig. 6K), suggesting that Aβ_ice_ did not modulate APP processing in vivo. These data show that inoculation of different variants of Aβ in APP_swe_/PS1_dE9_ AD mouse model resulted in similar levels of APP with similar production of Aβ.

### Aβ_ice_ reduces Tau pathology

**Fig. 7:**
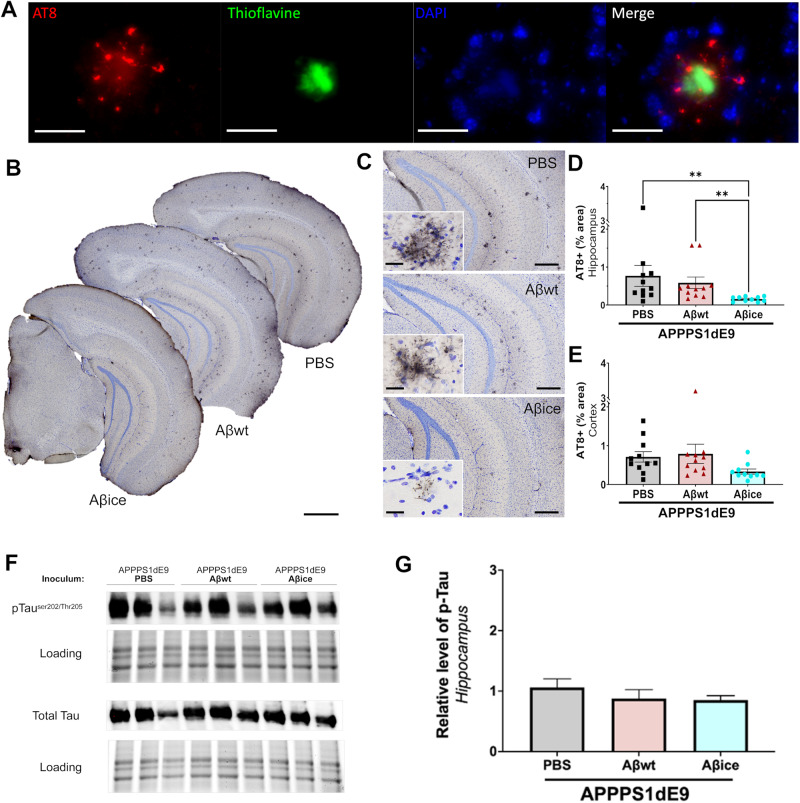
Aβ_ice_ reduces Tau pathology. **A** Representative images of AT8-Thioflavine S double labeling showing AT8-positive neurites surrounding an amyloid plaque (Thioflavine). **B** Representative images of AT8 immunolabeling showing neuritic plaques in the brain of APP_swe_/PS1_dE9_ mice after PBS, Aβ_wt_ or Aβ_ice_ inoculation in the dentate gyrus. **C** Representative images of Tau-positive neuritic plaques in the hippocampus. Magnified views showed no difference in the morphology of neuritic plaques between groups. Quantification of overall AT8-labeled phospho-Tau (percentage of AT8-positive area) revealed decrease of AT8-positive area in the hippocampus (**D**) of Aβ_ice_-inoculated APP_swe_/PS1_dE9_ mice compared with PBS- (**p = 0.002) and Aβ_wt_-inoculated (**p = 0.003) APP_swe_/PS1_dE9_ mice. AT8 staining was not significantly different in the cortex of the different groups (**E**). nAPP/PS1_PBS_ = 10, n_Aβwt_ = 11, n_Aβice_ = 12 mice. **p < 0.01. Data are shown as mean ± s.e.m. Scale bars: **A** 25 µm; B: 500 µm; **C** main images = 100 µm, Insets = 20 µm. **F** Western-blot analysis of phosphorylated Tau (AT8 antibody) and total Tau in hippocampal lysates of APP_swe_/PS1_dE9_ mice after PBS, Aβ_wt_ or Aβ_ice_ inoculation. The phosphorylated Tau and total Tau run at an apparent molecular size of 58 kDa. **G** Semi-quantification of phosphorylated Tau level in the hippocampus of inoculated APP_swe_/PS1_dE9_ mice. The phosphorylated form was normalized versus the total form of Tau. The level of pTau was similar between groups (p = 0.10; nAPP/PS1_PBS_ = 3, n_Aβwt_ = 3, n_Aβice_ = 3 mice). Data are shown as mean ± s.e.m.

At 4 mpi, we detected AT8-positive neurites surrounding amyloid plaques (Fig. 7A) in the hippocampus (Fig. 7B, C) and cortex (Fig. 7B) of all inoculated APP_swe_/PS1_dE9_ mice. These lesions were not detected by omitting the primary AT8 antibody (Supplementary Fig. 2) and were classified as neuritic plaques in APP_swe_/PS1_dE9_ mice [16, 32] as well as in other mouse models of AD as APP-KI and 5X-FAD [33], APP_swe_/PS1_L166P_ [34] or Tg2576 [35]. Remarkably, the quantification of overall AT8-labeled phospho-Tau significantly decreased in the hippocampus of Aβ_ice_-inoculated APP_swe_/PS1_dE9_ mice compared with animals inoculated with PBS or Aβ_wt_ (Fig. 7D). The amount of AT8-positive tau lesions in the cortex was similar between groups (Fig. 7E). Then, we assessed the level of AT8-positive phosphorylated Tau and total Tau (Fig. 7F) in hippocampal lysates. We did not find any significant difference between groups for AT8-positive Tau/total tau (Fig. 7G) nor for AT8-positive (not shown) or total Tau (not shown). Previous studies have suggested that neuritic-like plaques in the hippocampus are actually astrocytes containing polyglucosan bodies [36–38]. To rule out this possibility, we performed a triple labeling using AT8, GFAP for astrocytes and DAPI for cell nuclei. This confirmed that AT8-positive lesions were not detected within astrocytes **(**Supplementary Fig. 2**)**. Furthermore, using periodic acid Schiff (PAS) staining that detect polyglucosan bodies in astrocytes [37], we did not detect any strongly positive PAS staining within astrocytes (Supplementary Fig. 2).

### Aβ_ice_ reduces detrimental synaptic engulfment by microglia

**Fig. 8:**
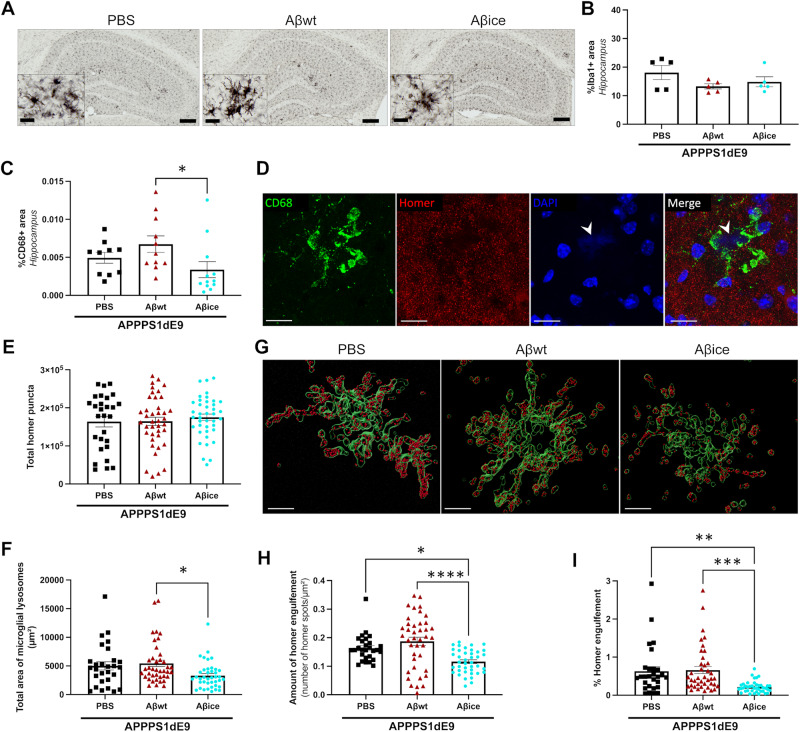
Reduction of synaptic microglial engulfment in Aβ_ice_-inoculated APP_swe_/PS1_dE9_ mice. **A** Representative images of Iba1 immunolabeling showing microglia in the hippocampus of APP_swe_/PS1_dE9_ mice after PBS, Aβ_wt_, or Aβ_ice_ inoculation. Scale bar: main images = 100 µm, Insets = 20 µm. **B** Quantification of Iba1 staining revealed similar microglial density in the hippocampus at 4mpi (p = 0.383; Kruskal-Wallis with Dunn’s multiple comparisons). n_APP/PS1-PBS_ = 5, n_Aβwt_ = 5, n_Aβice_ = 5 mice. **C** Quantification of total CD68 in the hippocampus showed an increased lysosomal microglia density in Aβ_wt_-inoculated APP_swe_/PS1_dE9_ mice compared to Aβ_ice_-inoculated APP_swe_/PS1_dE9_ mice (p = 0.019). No difference was observed between PBS- and Aβ_ice_-inoculated APP_swe_/PS1_dE9_ mice (p = 0.383). Kruskal-Wallis with Dunn’s multiple comparisons. n_APP/PS1-PBS_ = 10, n_Aβwt_ = 11, n_Aβice_ = 12 mice. **D** Co-immunolabeling of lysosomal microglia (CD68, green), post-synaptic (Homer, red) and DAPI (blue) markers. Scale bars= 20 µm **E** Quantification of total Homer puncta revealed no difference between groups. **F** Quantification of CD68-positive areas revealed a decrease of microglial lysosomes area in Aβ_ice_-inoculated APP_swe_/PS1_dE9_ mice compared to Aβ_wt_-inoculated APP_swe_/PS1_dE9_ mice (p = 0.01) but no significative difference with PBS-inoculated APP_swe_/PS1_dE9_ mice. **G** CD68-positive lysosomes (green) and Homer spot (red) are 3D-reconstructed to assess phagocytosis of post-synaptic compartment in APP_swe_/PS1_dE9_ mice after PBS, Aβ_wt_, or Aβ_ice_ inoculation. Scale bars= 10 µm. **H** Quantification of Homer spot inside CD68-positive lysosomes revealed a decreased amount of homer microglial engulfment in Aβ_ice_-inoculated APP_swe_/PS1_dE9_ mice compared to PBS- (p = 0.014) and Aβ_wt_-inoculated APP_swe_/PS1_dE9_ mice (p < 0.0001). **I** Quantification of the number of Homer spot engulfed versus the total Homer puncta revealed a decreased microglial engulfment of homer marker in Aβ_ice_-inoculated APP_swe_/PS1_dE9_ mice compared to PBS- (p = 0.003) and Aβ_wt_-inoculated APP_swe_/PS1_dE9_ mice (p = 0.0003). Kruskal-Wallis with Dunn’s multiple comparisons. n_APP/PS1-PBS_ = 7, n_Aβwt_ = 10, n_Aβice_ = 10 mice. *p < 0.05, **p < 0.01, ***p < 0.001, ****p < 0.0001. Data are shown as mean ± s.e.m.

We investigated the impact of Aβ seeds inoculation on microglia-associated neuroinflammatory response. First, we assessed the overall density of microglia in the hippocampus by performing Iba1 staining, a pan-microglial marker (Fig. 8A). Microglia clusters were mainly found surrounding amyloid plaques **(**Supplementary Fig. 3**)**. Abundant activated microglia around amyloid plaque were characterized by spherical cell body with dystrophic ramifications and were similar between groups (Fig. 8A). Quantification of Iba1 immunoreactivity showed similar microglia density in the hippocampus of inoculated APP_swe_/PS1_dE9_ mice (Fig. 8B). Then, to address microglial activation, we used an anti-CD68 antibody that stains a lysosomal protein expressed at high levels by activated microglia and at low levels by resting microglia. CD68 immunoreactivity was found inside Iba1-positive microglial cell body (Fig. S3). Quantification of the overall CD68 showed a lower immunoreactivity in Aβ_ice_-inoculated mice compared to Aβ_wt_-inoculated mice, but no difference with PBS-inoculated animals (Fig. 8C). Previous studies have shown that CD68+ microglial lysosomes engulf synapses [27]. Given the loss of synapses detected in Aβ_ice_-animals, we wondered whether microglial synaptic engulfment could be an indirect target of Aβ_ice_-mediated protection. We performed a double labelling of CD68 microglia lysosomes and post-synaptic Homer markers (Fig. 8D–I) [27]. Using confocal microscopy, we recorded images of microglia surrounding amyloid plaques, which generate blue autofluorescence (DAPI positive) (Fig. 8D). CD68-positive lysosomes area were lower in Aβ_ice_-inoculated mice compared to Aβ_wt_-inoculated mice (Fig. 8F). This suggests a reduced phagocytic activity of microglia close to amyloid plaques in Aβ_ice_-mice. The total number of post-synaptic homer puncta in the images was similar in the different groups (Fig. 8E). The 3D reconstruction of CD68-positive surfaces allowed us to quantify the number of Homer spots into microglia lysosomes (Fig. 8G–I). Aβ_ice_-inoculated mice displayed reduced amount of homer puncta within lysosomes compared to PBS- and Aβ_wt_-inoculated mice (Fig. 8H, I). This suggests a decreased amount of post-synaptic engulfment in Aβ_ice_-inoculated mice.

We also quantified astrocytic reactivity using GFAP staining. We did not detect any differences between the different groups of animals (Supplementary Fig. 3).

## Discussion

### Aβ_ice_ can participate to the synaptoprotection induced by the APP Icelandic mutation

The Icelandic mutation (A673T, Ice) protects against AD and age-related cognitive decline [3]. A widely advanced hypothesis to explain this protective effect is that APP_ice_ leads to reduced Aβ production [3–6] and thus reduces Aβ-dependent synaptotoxicity. In our cortical neuron studies, neurons over-expressing APP_ice_ did not display synaptic deficits while neurons over-expressing APP_wt_ or APP_swe_ did. At first sight, this supports the initial hypothesis of reduced toxic-Aβ production by APP_ice_. However, we found that although the over-expression of APP_wt_ (but not of APP_ice_) in neurons increases synaptotoxicity, the amount of Aβ was only slightly reduced in protected APP_ice_ neurons. To test the hypothesis that produced Aβ_ice_ is not synaptotoxic, we applied equal “high” concentrations of Aβ_wt_ or Aβ_ice_ on acute mouse hippocampal slices for long-term potentiation (LTP) electrophysiological experiments, as well as on cultured cortical neurons to assess spine morphology. As expected, we observed toxic effects of Aβ_wt_ in both models. Interestingly, Aβ_ice_ did not induce any alterations on LTP or spine morphology. Thus, we demonstrated that Aβ loses its toxicity when it bears the Icelandic mutation. Moving one step forward, we outlined that application of Aβ_ice_ on primary APP-cortical neurons overproducing endogenous Aβ led to an increase in mature spine density. Thus, Aβ_ice_ can prevents Aβ-induced spine density decrease. In addition to other possible protective effect of APP_ice_, the ability of Aβ_ice_ to rescue synaptotoxicity induced by Aβ can explain protection of the Icelandic mutation carriers.

To further investigate this hypothesis in vivo, we exposed the hippocampus of a commonly used mouse model of amyloidosis to Aβ_ice_. Interestingly, this rescued spatial memory measured 4 months post-inoculation. One can not rule out that an acute protective effect of Aβ_ice_ was maintained for 4 months, but we however investigated other long-term changes occurring in Aβ_ice_-inoculated animals to explain the long-term in vivo protective effect. First, Aβ_ice_ inoculation increased synaptic density as compared with Aβ_wt_ and also PBS-inoculated animals and restored synaptic density to the level observed in wild-type animals. This suggests a long-term protective effect of Aβ_ice._ We also found a reduction of CD68 lysosomal markers in microglia surrounding Aβ plaques of Aβ_ice_ animals as well as a reduction of synaptic engulfment by microglia lysosomes. This suggests that the synaptoprotection induced by Aβ_ice_ is at least partly associated with changes of microglia-related synaptic phagocytosis close to amyloid plaques.

### Aβ_ice_ does not modulate amyloid plaque load in vivo

Modulation of APP processing is the main hypothesis explaining the reduced Aβ production in Icelandic careers. This hypothesis is clearly demonstrated in numerous studies on cell models. Other rising strategies with APP_A673T_ variant based on genome-editing approaches [39, 40] will likely clarify in the near future whether or not the Icelandic mutation retains its effects on APP processing even in vivo. In our inoculated Aβ_ice_ mouse model, both APP processing products (β-CTF/C99 and α-CTF/C83) were not modulated suggesting that Aβ_ice_ targets mechanisms that are independent from APP processing. It is not surprising that the treatment of our mouse model of amyloidosis with Aβ_ice_ did not result in any alterations of APP processing, since Aβ_ice_ injected in the animal models is not the APP_A673T_ (*i.e*., the substrate of the β-secretase activity) but Aβ_ice_ (*i.e*., the post-processing Aβ fragment of APP_A673T_).

It is well established that Aβ toxicity arises from pathological/excessive amounts of Aβ as well as from abnormal Aβ aggregation. Several studies in animal models have shown that intracerebral infusion of Aβ-positive solutions induces build-up of Aβ deposits in their host several months after the infusion [12–17]. Mechanistic models explaining prion diseases have been largely used to interpret this result. They suggest that in presence of preformed amyloid seeds, newly produced non-β-sheet monomers can change their conformation to assemble into novel aggregated amyloid structures thus inducing a self-propagating process [29]. Following these models, our initial hypothesis on beneficial effects of Aβ_ice_ is that it would modulate Aβ aggregation within our APP_swe_/PS1_dE9_ mice. Indeed, previous studies based on fibrillization assays have shown that Aβ_ice_ decreases Aβ aggregation [4, 6, 41]. Also, our fibrillogenesis assay of synthetic Aβ_1-42_ exposed to Aβ_ice_ showed a delay of Aβ aggregation kinetics. However, our in vivo experiments highlighted that a single inoculation of Aβ_ice_ did not change cerebral amyloid plaque load four months post-inoculation. Thus Aβ_ice_ does not impact aggregation of Aβ in vivo. This does not rule out possible not-detected effects on soluble forms of Aβ.

### Aβ_ice_ lowers Tau pathology

Studies in animal models have shown that intracerebral infusion of Aβ-positive solutions can worsen Tau pathology [16]. Here, unexpectedly, we reported that Aβ_ice_ inoculation lowers Tau pathology. Our study is the first one to show in vivo a positive impact of Aβ species inoculation on phospho-Tau aggregates. This effect was not associated with changes of amyloid loads in the inoculated animals, nor with changes of APP processing. At this stage, we cannot decipher the origin of this change, but our results are in line with recent publications suggesting that fragments of Aβ with a mutation on the second amino acid can modulate Tau species in the brain [42]. Aβ is known to stimulates tau phosphorylation at the synaptic level [43, 44]. Also studies in animal models have suggested that Tau can induce synaptic deficits [27, 45, 46]. One possible explanation for our overall results is that Aβ_ice_ interacted with tau phosphorylation processes and that the reduced tau phosphorylation at the synaptic level was associated with a reduction of synapses phagocytosis. Further studies will have to be performed in the future to investigate this hypothesis.

### Towards therapeutic approach stemming from protective genetic variants of human Aβ

Several therapeutic approaches have been proposed against AD and many therapies targeting Aβ as γ-secretase inhibitors, β-secretase inhibitors, α-secretase modulators, aggregation inhibitors, metal interfering drugs, or drugs that enhance Aβ clearance are under investigation [47]. In particular, high hopes are put in recent anti-Aβ immunotherapies that can delay disease evolution [48]. Approaches based on protective variants of APP are intriguing. Here we showed that Aβ_A2T_ mutation is protective in vivo. Another variant (APP_A673V_, Aβ_A2V_), localized on the same amino-acid as A2T, was also shown to be protective [42, 49]. This latter mutation leads to Alzheimer’s disease in the homozygous state and is protective in heterozygous state [50]. It induces a reduction of fibrillogenic properties of wild-type Aβ in the presence of Aβ_A2V_ [50]. Thus potential therapies were developped using small synthetic peptides limited to the first amino acids of Aβ_A2V_ [49]. After 20 weeks of intranasal administration every other day, these peptides could lower amyloid plaque load and increase synaptic integrity [49]. The effects of peptides derived from Aβ_A2V_ on synapses remind those that we found with Aβ_A2T_. Also, as A2T, mutation on A2V seems to mitigate tau pathology [42]. Further comparisons between the A2T and A2V “protective” variants are now required to better understand their protective mechanisms of action. One of the remarkable difference between our study and previous results is that, unexpectedly, a single inoculation of Aβ_A2T_ had a long term impact four months after inoculation. In other proteionopathies, as in the case of prion diseases, therapeutic strategies based on a single inoculation of a innocuous variant form (referred as “anti-prion”) of a pathological protein has been proposed [51]. The rationale for the “anti-prion” hypothesis, is that that the innocuous variant could compete with prion substrate to inhibit prion replication and lower the pathology. This concept is inspiring for our results even if at this stage we can not propose a mechanisms for Aβ_ice_ action and in particular we did not detect a reduction of amyloid plaque load.

In summary, we showed that Aβ_ice_ rescues synaptotoxicity induced by Aβ. This can be a novel mechanisms explaining protection of subjects carrying the Icelandic mutation. More interestingly, a single inoculation of Aβ_ice_ induces long-term protection from synaptic damage associated with a rescue of spatial memory performance. The reduction of synaptic damage is associated with a lower phagocytosis of synapses by lysosomes and reduction of Tau pathology in a mouse model of amyloidosis. These results suggest that a single sporadic event as Aβ_ice_ inoculation can induce and propagate a protective phenotype that is maintained several months after the event and could be used as a new therapeutic approach to counteract Aβ-mediated toxicity.

## Supplementary information

Supplementary Figures

Supplementary Materials

Supplementary Table 1

## Data Availability

The data that support the findings of this study are available from the corresponding author, upon request.
