# Supplementary material for: Transmissible long-term neuroprotective and pro-cognitive effects of 1–42 beta-amyloid with A2T icelandic mutation in an Alzheimer’s disease mouse model
Source: Mol Psychiatry. 2024 Jun 14;29(12):3707–21. doi: 10.1038/s41380-024-02611-8 (PMC11609088; doi:10.1038/s41380-024-02611-8)

SUPPLEMENTARY FIGURES

Transmissible long-term neuroprotective and pro-cognitive effects of 1-42 beta-amyloid with A2T icelandic mutation in an Alzheimer's disease mouse model

Marina Célestine[1,2], Muriel Jacquier-Sarlin[3], Eve Borel[3], Fanny Petit[1,2], Fabien Lante[3], Luc Bousset[1,2], Anne-Sophie Hérard[1,2], Alain Buisson[3], Marc Dhenain[1,2]

Author affiliations:

[1] Université Paris-Saclay, CEA, CNRS, Laboratoire des Maladies Neurodégénératives, 18 Route du Panorama, F-92265 Fontenay-aux-Roses, France.

[2] Commissariat à l'Energie Atomique et aux Energies Alternatives (CEA), Direction de la Recherche Fondamentale (DRF), Institut de Biologie François Jacob, MIRCen, 18 Route du Panorama, F-92265 Fontenay-aux-Roses, France

[3] Univ. Grenoble Alpes, Inserm, U1216, Grenoble Institut Neurosciences, GIN, 38000 Grenoble, France

Correspondence to: Marc Dhenain, DVM, PhD
Université Paris-Saclay, CEA, CNRS, Laboratoire des Maladies Neurodégénératives, MIRCen, 18 Route du Panorama, F-92265 Fontenay-aux-Roses, France ;
E-mail: Marc.Dhenain@cnrs.fr

Figure S1.

Efficacy of basal synaptic transmission measured thanks to input/output (I/0) curves and paired pulse facilitation ratio (PPR).

A. Input/output (I/O) curves. Curves were constructed by plotting mean fEPSPs slopes + SEM as a function of stimulation intensity. There was not significant differences between the different groups (two way ANOVA, Aβ conditions x stimulation intensity interaction ($F (18, 170) = 0.17$, $p>0.9$), n = at least 5 different mice per condition). **B.** Paired pulse facilitation ratio (PPR). Curves were constructed by plotting PPR + SEM as a function of inter-pulse interval. There was not significant differences between the different groups (two way ANOVA, Aβ conditions x inter-pulse interval interaction ($F (12, 119) = 0.5245$, $p=0.9$), n =at least 5 different mice per condition.

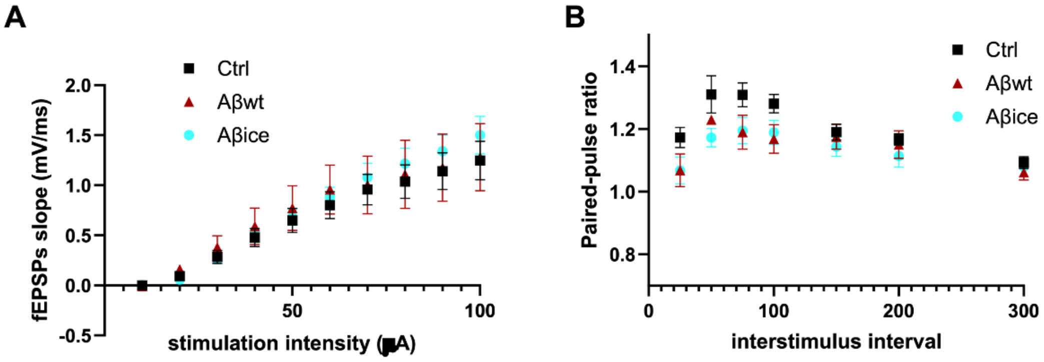

Figure S2.

Neuritic-like plaques in the hippocampus are not astrocytes containing polyglucosan bodies

A-B. AT8 labeling including the primary antibody (**A**) and without the primary antibody (**B**). The neuritic plaques were not detected anymore, thus suggesting that AT8-labeling was specific. **C.** Double staining with GFAP for astrocytes and PAS that detects polyglucosan bodies. The amyloid plaque cores are slightly PAS positive, but astrocytes are not positives. **D-E.** Triple labeling using AT8 (green), GFAP for astrocytes (red) and DAPI (blue) for cell nuclei. AT8-positive lesions were not detected within astrocytes.

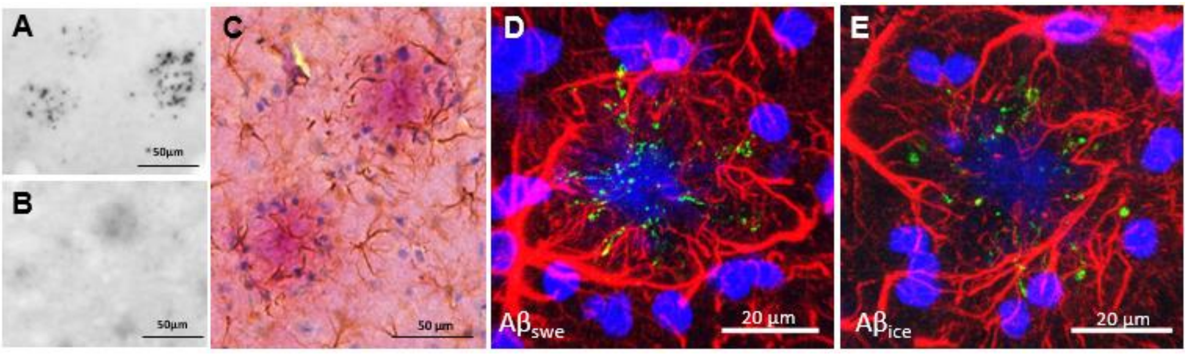

Figure S3.

Microglia and astrocyte labelling in the hippocampus of inoculated APP/PS1$_{dE9}$ mice

A. Co-immunolabeling using Iba1 for astrocytes (green), 4G8 for amyloid plaque (red) and DAPI (blue) for cell nuclei. Iba1-positive clusters were found around amyloid plaque. Scale bar = 30 µm **B**. Co-immunolabeling using Iba1 for astrocytes (green), CD68 for lysosomal microglia (red). DAPI (blue) was used for the labelling of amyloid plaque autofluorescence . Overlap of Iba1 and CD68 labelling were found surrounding amyloid plaques. Scale bar = 20 µm **C.** Representative images of GFAP immunolabeling showing astrocytes in the hippocampus of APP$_{swe}$/PS1$_{dE9}$ mice after PBS, Aβ$_{wt}$, or Aβ$_{ice}$ inoculation. Scale bars: main images = 100 µm, Insets = 20 µm **D.** Quantification of GFAP staining revealed similar astrocyte density in the hippocampus at 4mpi (p=0.6808 ; Kruskal-Wallis with Dunn's multiple comparisons). n$_{APP/PS1-PBS}$=5, n$_{Aβwt}$=5, n$_{Aβice}$=5 mice.

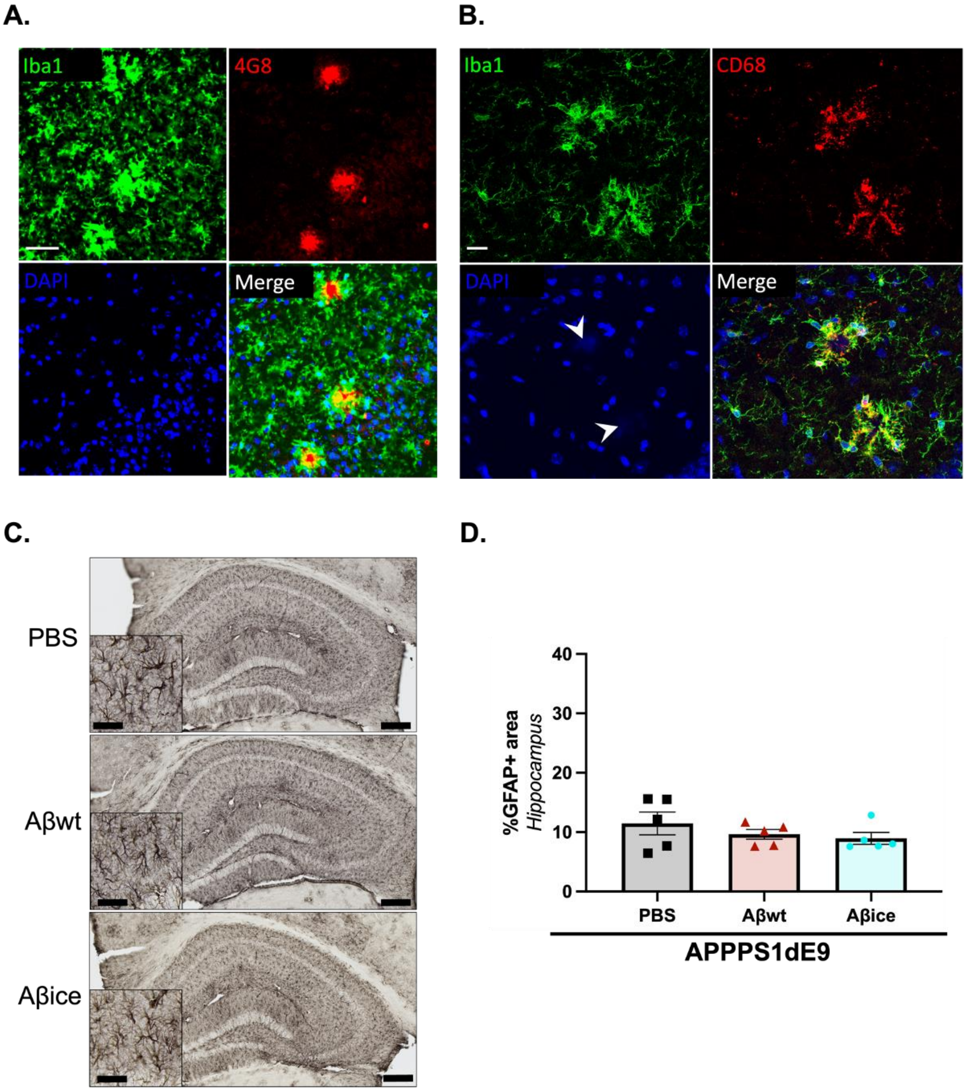

Supplement: Supplementary file 1 — Supplementary Figures [file 41380_2024_2611_MOESM1_ESM.pdf]
