# Supplementary material for: Transmissible long-term neuroprotective and pro-cognitive effects of 1–42 beta-amyloid with A2T icelandic mutation in an Alzheimer’s disease mouse model
Source: Mol Psychiatry. 2024 Jun 14;29(12):3707–21. doi: 10.1038/s41380-024-02611-8 (PMC11609088; doi:10.1038/s41380-024-02611-8)
Supplement: Supplementary file 2 — Supplementary Materials [file 41380_2024_2611_MOESM2_ESM.pdf]

SUPPLEMENTARY MATERIALS

Transmissible long-term neuroprotective and pro-cognitive effects of 1-42 beta-amyloid with A2T icelandic mutation in an Alzheimer's disease mouse model

Marina Célestine[1,2], Muriel Jacquier-Sarlin[3], Eve Borel[3], Fanny Petit[1,2], Fabien Lante[3], Luc Bousset[1,2], Anne-Sophie Hérard[1,2], Alain Buisson[3], Marc Dhenain[1,2]

Author affiliations:
[1] Université Paris-Saclay, CEA, CNRS, Laboratoire des Maladies Neurodégénératives, 18 Route du Panorama, F-92265 Fontenay-aux-Roses, France.
[2] Commissariat à l'Energie Atomique et aux Energies Alternatives (CEA), Direction de la Recherche Fondamentale (DRF), Institut de Biologie François Jacob, MIRCen, 18 Route du Panorama, F-92265 Fontenay-aux-Roses, France
[3] Univ. Grenoble Alpes, Inserm, U1216, Grenoble Institut Neurosciences, GIN, 38000 Grenoble, France

Correspondence to: Marc Dhenain, DVM, PhD
Université Paris-Saclay, CEA, CNRS, Laboratoire des Maladies Neurodégénératives, MIRCen, 18 Route du Panorama, F-92265 Fontenay-aux-Roses, France
E-mail: Marc.Dhenain@cnrs.fr

SUPPLEMENTARY METHODS

Primary cultures of cortical neurons

Mouse cortical neurons were cultured from 14- to 15-day-old OF1 embryos (Janvier) as described previously (1). After extraction of the embryonic brains, the cerebral membranes were removed and the cortices were dissected, mechanically dissociated and cultured in Dulbecco's Modified Eagle's Medium (DMEM) supplemented with 5% horse serum, 5% foetal bovine serum (FCS) and 1 mM glutamine (all from Sigma) on 24-well plates (Falcon; Beckton Dickinson) for biochemical experiments.

Neurons were seeded on 35 mm glass-bottom dishes (MatTek) for confocal experiments. All plates, dishes, and coverslips were coated with 0.1 mg/ml poly-D-lysine and 0.02 mg/ml laminin (Sigma). Cultures were maintained at 37°C in a humidified atmosphere containing 5% CO_2/95% air. The day before the experiments, cells were washed in DMEM. Treatments were performed on neuronal cultures at 14–15 DIV

Plasmids

cDNAs of WT human APP_{695} (this vector was a gift from Dr Rémi Sadoul, Grenoble Institute of Neuroscience) was cloned into pmcherry-N1 vector (Snapgene) using the BamHI and AgeI restriction sites. Then, using overlapping PCR and the In-Fusion Cloning Kit (Takara) we generated

the Swedish mutant APP_{swe}-mCherry (N595K, L596M) and APP_{ice}-mCherry (A598T) plasmids as described by manufacturer's instructions. All constructions in pmCherry vector were verified by sequencing.

Neuronal transfection

Transfections were performed on cortical neuron cultures after 12 DIV with calcium phosphate precipitation. Growth medium (DMEM and sera) was removed and kept at 37°C until the last step of transfection. Cells were washed in DMEM and incubated for 30 min in DMKY buffer containing the following: 1 mM kynurenic acid, 0.9 mM NaOH, 0.5 mM HEPES, 10 mM $MgCl_2$ and phenol red 0.05%, pH 7.4. Then, 3 µg of the plasmids containing the APP_x-mCherry and LifeActin-GFP (LA-GFP), a peptide which specifically binds filamentous actin (2), were mixed with $CaCl_2$ (120 mM) in HBS containing the following: 25 mM HEPES, 140 mM NaCl, and 0.750 mM Na_2HPO_4, pH7.06) and left for 15 min to precipitate the DNA. Plasmids were then applied to cells for 60 min.

Production of recombinant Aβ peptides

To make the plasmids for the fusion protein Aβ(His) of wild-type human β-amyloid 1-42 protein ($Aβ_{wt}$) or $Aβ_{ice}$ mutant (A2T), the cDNA containing the sequence for human $Aβ_{1-42}$ was obtained from synthetic oligonucleotides (Sigma, Lyon, France) - containing a Nde1 restriction site as forward primers and a PspXI restriction site as reverse primers - using overlapping PCR. PCR products were then cloned into a pet28a-vector (Novagen, Paris, France) and subsequently constructed as various mutant HIS-$Aβ_{1-42}$ expressing plasmids (pet28a-AβHis or pet28a-Aβ(His)ice: A2T). The resulting plasmids were verified by sequencing. Escherichia Coli BL21 (DE3) was transformed with the fusion protein plasmids and a single colony was chosen to grow in a 250 ml starter culture in Luria broth (LB medium) overnight (ON) at 37°C. The next day, 10 ml of culture was diluted in 1L LB culture medium. When the culture reached an OD_{600nm} of 0.8, isopropyl-beta-D-thiogalactopyranoside (IPTG) was added to 1 mM for induction. The culture was grown for an additional 4 hours and the cells harvested by centrifugation at 4000g for 20 min. The pellet was re-suspended in 10 ml ice-cold PBS and lysed by sonication at ice-cold temperature. The cell extract was then centrifuged at 20,000g for 15 min at 4°C. The pellet was re-suspended in 10 ml of 8 M urea in sterile culture PBS and sonicated as previously described before centrifugation at 20,000g for 15 min at 4°C. The supernatant (5 ml) was diluted with 15 ml of binding buffer (PBS with 10 mM imidazole at pH 8.0). Before affinity purification using nickel-nitriloacetic acid (Ni-NTA) column (3 ml of protino Ni-NTA Agarose from Macherey Nagel), samples were filtered on 0.45 µm. The Ni-NTA column was equilibrated with binding buffer prior to loading the sample on the column. Then the column was washed with the washing buffer (PBS with 30 mM imidazole at pH 8.0) with 5-10

column volumes. The protein was then eluted with the elution buffer (PBS with 500 mM imidazole at pH 7.4). The absorbance at 280 nm was used to monitor the elution. The concentration of the fusion proteins was estimated by comparing the band intensity of the protein on SDS-PAGE with known quantity of BSA (Sigma). A final concentration of 100 µM was obtained and aliquots were stored at -80°C. Aliquots from all subsequent purification steps were analysed by SDS-PAGE, and the identity of $Aβ_{1-42}$ and mutants was verified by western blots using 4G8 monoclonal antibodies against Aβ sequence (4G8).

Endotoxin assay

Endotoxin content of Aβ solutions was detected using a kinetic Limulus amebocyte lysate (LAL) chromogenic endotoxin quantitation kit (Thermo Scientific). In brief, 50 µM of Aβ solution was prepared in sterile culture solution of saline phosphate buffer pH 7.4 (PBS) and was transferred to a sterile 96-well plate pre-warmed to 37°C. LAL (0.1 ml, room temperature, RT) was quickly added to each well. Detection relied on standards ranging from 0.10 to 1 EU/ml and on positive and negative controls, supplied in the kit, that were performed at the same time as the samples. Endotoxin concentrations were determined by measuring kinetic absorbance at 405 nm at 37°C following the instructions of the manufacturer, in a Spark plate reader (Tecan).

Lentivirus production for Aβ production measurements

To generate infectious lentiviral particles, sequences of various human APP695 mutants were cloned into pLenti-C-mCherry vector using the BamH1 and pmeI restriction sites. This vector was a gift from Dr Christophe Bosc (Grenoble Institute of Neuroscience). cDNAs of WT human APP_{695} , human Swedish mutant APP_{swe} (N595K, L596M) and human APP_{ice} (A598T) were obtained using overlapping PCR on APPx-mCherry plasmid. PCR fragments were cloned into pLenti-C-mCherry vector using the BamH1 and pmeI restriction sites and the In-Fusion Cloning Kit (Takara) as described by manufacturer's instructions. All constructions in pLenti-C-mCherry vector were verified by sequencing. psPAX2 is a packaging plasmid encoding HIV-1 gag/pol sequences under the control of a SV40 promoter (Addgene plasmid # 12259). pCMV-VSV-G is an envelope-expressing plasmid encoding for VSV-G glycoprotein under the control of a CMV promoter (Addgene plasmid # 8454).

Lentiviruses were produced in HEK293T cells from various pLenti-APPx-mCherry plasmids. For the virus production, HEK293T cells were transfected using Ca^{2+}- phosphate in cell culture dishes (100 x 15 mm) with a given lentiviral plasmid and the two helper plasmids psPAX2 and pCMV-VSV-G. Six hours after transfection, the culture medium (DMEM glutamax containing 10% heat-inactivated FCS and 1% penicillin-streptomycin) was changed to remove transfection reagent in

the conditioned medium to which the virus is secreted. Then, 48 hours after transfection conditioned medium was spun at 250g for 5 min at 4°C before being collected and filtered using a 0.45 µm sterile filter (Sarstedt, Nuembrecht, Germany). Then virus particles were pelleted by ultra-centrifugation for 2 hours at 4°C and 20,000 rpm in a Beckman SW32Ti swinging bucket rotor. Supernatant was discarded and virus was suspended in PBS (X100 concentrated according to the initial volume of supernatant) and aliquots of the viral solution were snap-frozen in liquid nitrogen before storage at -80°C until use. Lentivirus titration was performed by FACS analysis after viral transduction of HEK293T and was estimated around 2 10^8 UI/mL.

For viral transduction, lentiviral solutions were diluted (1:10) in culture medium and 50 µL of the diluted preparation were added to the culture medium of a 24-well plate (for human neuronal cultures). Cell culture supernatant or lysates were harvested 72 hours post-infection.

Aβ production measurements *by ELISA assay*

To assess the level of « total » Aβ (secreted into the medium or produced in cell lysate), after 72 hours infection of cortical neurons with lentivirus producing various APP_X mutants we performed an ELISA assay. For this assay, samples (200 µl of cell culture medium or lysates corresponding to 150 µg of proteins) and standards were incubated overnight (ON) at 4°C in a maxisorb 96-well plate (Nunc). The Aβ (1–42 aa) standards were prepared using synthetic Aβ ranging from 0.1 to 2 µg/ml. The plates were then aspirated and blocked with 3% bovine serum albumin (BSA) in PBS buffer for at least 1 hour at 37°C. The samples and standards were added to the plates and incubated at RT for 1 hour. The 6E10 antibodies diluted to 0.2 µg/ml in blocking buffer was incubated in the wells for 1 hour at room temperature RT. The plates were washed three times with wash buffer (0.05% Tween 20 in PBS buffer containing 1% BSA). Horseradish peroxidase conjugated anti mouse antibodies (Jackson Laboratories), diluted 1:5000 in blocking buffer, was added to the wells for 1 hour at 37°C. Then the plates were washed three times with wash buffer and once with PBS. The colorimetric substrate, Ultra TMB-ELISA (Pierce), was added and allowed to react for 15 min, after which the enzymatic reaction was stopped with addition of 1 M H_2SO_4. Reaction product was quantified using a Molecular Devices Vmax spectrophotometer measuring the difference in absorbance at 405 nm and 650 nm. The low end sensitivity of this assay is 50 ng/ml (14 nM; data not shown). The efficient of infection was controlled by western blot on 15% SDS-PAGE using Y188, a rabbit monoclonal antibody (against the C-terminus of APP, Abcam) and a mouse monoclonal actin antibody (Sigma) as loading control.

Quantification and morphological characterisation of dendritic spine density *in vitro*

Neurons were visualized using a Nikon Ti C2 confocal microscope with a Nikon 60X water-immersion objective and NIS-Elements software (Nikon, Melville, NY, USA). Excitation of GFP and

mCherry fluorophores was performed with an argon laser at 488 nm (emission filtered at 504-541 nm) and at 543 nm (emission filtered at 585-610 nm) respectively. Images (1024x1024 pixels) were acquired as Z-stacks (tridimensional section) with 0.3 µM per step. The acquired images were then deconvoluted using AutoQuantX3 software (Media Cybernetics, Abingdon, Oxon, UK). For analysis of spines, serial image files corresponding to z-stacks of 20–30 optical sections per dendritic segment were directly processed with NeuronStudio, a software package specifically designed for spine detection and analysis (https://biii.eu/neuronstudio, CNIC – Mount Sinai School of Medicine). Voxel size was 0.3x0.3x0.3 µm. After modeling of the dendrite surface, protrusions with a minimum volume of 5 voxels, length of between 0.2 µm and 4 µm and a maximal width of 3 µm were retained as spines. Following default settings of the program and the empirical classification rule previously described (3), spines with a minimum head diameter of 0.35 µm and minimum head vs neck ratio of 1.1 were classified as mushroom spines. Non-mushroom spines with minimum volume of 10 voxels (0.040 μm^3) were classified as stubby spines. All other spines were considered thin.

Electrophysiology recordings

Horizontal brain slices containing the somatosensory cortex were prepared from 20 to 30 day-old OF1 mice. Mice were cervically dislocated and immediately decapitated. Their cortices were dissected out and 300 µm thick transverse slices were cut in ice-cold cutting solution (in mM: KCl 2.5, NaH2PO4 1.25, MgSO4 10, CaCl2 0.5, NaHCO3 26, Sucrose 234, and Glucose 11, saturated with 95% O2 and 5% CO2) with a Leica VT1200 blade microtome. After the dissection, slices were kept in oxygenated ACSF at 37±1°C for at least 1 h.

Slices were visualized in a chamber on an upright microscope with transmitted illumination and continuously perfused at 2 ml/min with oxygenated Artificial Cerebro-Spinal Fluid (ACSF in mM: 119 NaCl, 2.5 KCl, 1.25 NaH_2PO_4, 1.3 $MgSO_4$, 2.5 $CaCl_2$, 26 $NaHCO_3$, and 11 Glucose) at room temperature. Stimulating electrodes (bipolar microelectrodes) were placed in the stratum radiatum to stimulate the Schaffer collateral pathway. Field EPSPs (fEPSPs) were recorded in the stratum radiatum using a recording glass pipette filled with ACSF and were amplified with an EPC 10 Amplifier Patchmaster Multi-channel (HEKA Elektronik Dr. Schulze GmbH, Wiesenstrasse, Germany). Recordings were filtered at 1 kHz using the Patchmaster Multi-channel data acquisition software (HEKA Elektronik Dr. Schulze GmbH, Wiesenstrasse, Germany). The initial slope of the fEPSPs was measured to avoid population spike contamination. Input/output (I/O) curves characterizing basal glutamatergic transmission at CA3-CA1 synapses were constructed by plotting mean fEPSPs slopes + SEM as a function of stimulation intensity (10 to 100 µA). To assess short term plasticity, paired-pulse facilitation (PPF) was induced by paired-stimulation with an interpulse

interval from 25 to 300 ms. PPF was quantified by normalizing the slope of the second fEPSPs to the slope of the first one. For LTP experiments, test stimuli (0.2 ms pulse width) were delivered once every 15 s and the stimulus intensity was set to give baseline fEPSP slopes that were 50% of maximal evoked slopes. Slices that showed maximal fEPSP sizes < 1mV were rejected. Long-term potentiation (LTP) in the hippocampal CA1 region was induced by delivering two 100 Hz protocols (2 x 100 Hz) with an interval of 20 s to the Schaffer collateral/commissural pathway. Aβx peptides were added to the ACSF bath (final concentration of Aβx: 100 nM) 15 min prior to recording. 2 x 100 Hz was delivered after 15 min of stable baseline.

Dynamic Light Scattering

Aβ solutions [30 µM] were prepared by dilution in elution buffer (PBS with 500 mM imidazole at pH 7.4). After a centrifugation step (1,000g, 1 minute), particle sizes were characterized at ambient temperature in micro-volume quartz cuvettes (sample volume of 50 µL), thermostated at 22°C using a Zetasizer equipped with 532 nm wavelength laser source (Malvern Instrument Zetasizer, Software Ver. 7.11). The hydrodynamic radius was calculated using a dispersant refractive index of 1.330, a viscosity of 0.9540 cP, and expressed as size distribution number.

Electron microscopy

Aβ solutions (100 µl sample of 8 µM) were concentrated 10 fold by centrifugation at 50,000g for 10 minutes and suspended in MilliQ water. Assemblies were layered on glow discharged carbon coated 400 mesh copper grid, and stained with 1% uranyl acetate. The assemblies were observed under Jeol 1400 electron microscope at 80kV and 10K magnification. Images were recorded on Rios CCD camera (Gatan).

Thioflavin T (ThT) binding assay

The seeding properties of $A\beta_{ice}$ and $A\beta_{wt}$ has been evaluated *in vitro* with the well-characterized thioflavin dye binding assay as previously described in *Celestine et al*. (4). Briefly, 4 µM of synthetic Aβ42 (Covalb, France) were adjusted to a final ThT (Wako Chemical Industries Ltd, Osaka, Japan) concentration of 10 µM in phosphate buffer, pH 7.4. All seeding experiments were performed with 10% or 2% of freshly prepared $A\beta_{wt}$ and $A\beta_{ice}$ solutions added to the synthetic Aβ1-42 + ThT solution. Fibril formation was followed by monitoring ThT fluorescence with shaking at 37°C for 24 hours using a Hitachi F-2500 fluorometer (445 nm excitation and 485 nm emission filters). Fluorescence was determined by averaging the three readings and subtracting the ThT blank. For each independent experiment, the lag phase parameter was extracted from the best fit to a sigmoid function using the following equations :

F(t)=Fmax / (1+exp(Kelongation x (T1/2)-T)) and Tlag = T1/ 2 – ln(2/Kelongation).

The one-way ANOVA statistical analysis of lag time was performed using GraphPad Prism.

Transgenic mice

In vivo experiments involved the APPswe/PS1dE9 mouse model of amyloidosis (C57Bl/6 background) (5, 6). Aβ plaques can be detected as early as 4 months of age in these mice and increase in number and total area with age (5). This model expresses endogenous murine Tau protein isoforms and is not transgenic for any human Tau. At the time of the inoculation of $A\beta_{wt}$, $A\beta_{ice}$ or PBS, at 2 months of age, these mice did not have Aβ plaques. Animals were studied for 4 months after intracerebral inoculation of the peptides, *i.e* 4 months post-inoculation (mpi). Group sizes were, respectively, $n_{APP/PS1\text{-}A\beta ice}$=12, $n_{APP/PS1\text{-}A\beta wt}$=11, $n_{APP/PS1\text{-}pbs}$=10. Wild-type littermates injected with the PBS were used as controls for the behavioral tests ($n_{WT\text{-}pbs}$=10). All $APP_{swe}/PS1_{dE9}$ and WT littermates were born and bred in our facility (Commissariat à l'Energie Atomique, centre de Fontenay-aux-Roses; European Institutions Agreement #B92-032-02). Females were exclusively used in this study in order to optimize group homogeneity (Aβ plaque load is known to vary between males and females). Mice were injected during different inoculation sessions and each group was randomly inoculated at each session to avoid an "order of treatment" confounding effect. All animals were identified using increasing numbers based on their birth-date and randomly assigned to the experimental groups using a simple procedure: they were alternatively assigned, in increasing order, to the *APP/PS1-wt* (animal 1, 4, 7…), *APP/PS1-ice* (animal 2, 5, 8…) and *APP/PS1-pbs* groups (animal 3, 6, 9…). All experimental procedures were conducted in accordance with the European Community Council Directive 2010/63/UE and approved by local ethics committees (CEtEA-CEA DSV IdF N°44, France) and the French Ministry of Education and Research (A20_017 authorization), and in compliance with the 3R guidelines. Animal care was supervised by a dedicated in-house veterinarian and animal technicians. Humane endpoints concerning untreatable continuous suffering signs and prostrations were taken into account and not reached during the study. Animals were housed under standard environmental conditions (12-h light-dark cycle, temperature at 22 ± 1°C and humidity at 50%) with *ad libitum* access to food and water. The design and reporting of animal experiments were based on the ARRIVE reporting guidelines (7). Sample size was based on previous experiments for Aβ induction in $APP_{swe}/PS1_{dE9}$ mice after inoculation of human brain samples (estimated with significance level of 5%, a power of 80%, and a two-sided Mann-Whitney's test) (8) and increased to take into account uncertainties for new markers (Tau lesion load, memory and synaptic changes). No animals were excluded from the study. MC was aware of initial group allocation, but further analysis (memory evaluations and post-mortem studies) were performed blinded.

Stereotaxic surgery

Five-hundred micrograms/ml (~ 150 nM) of $A\beta_{wt}$ or $A\beta_{ice}$ solution were rapidly thawed out before stereotaxic injection. Two-month-old $APP_{swe}/PS1_{dE9}$ and WT littermates were anesthetized by an intraperitoneal injection of ketamine (1mg/10g; Imalgène® 1000, Merial) and xylazine (0.1mg/10g; 2% Rompun®, Bayer Healthcare). Local anesthesia was also performed by a subcutaneous injection of lidocaine at the incision site (1 µl/g; 0.5% Xylovet®, Ceva Santé Animale). Mice were placed in the stereotaxic frame (Phymep, France) and bilateral injections of synthetic peptides were performed in the dentate gyrus (antero-posterior -2 mm, dorso-ventral 1.8 mm, lateral +/- 1 mm from bregma). Two µl/site of sample were administered using 34-gauge needles and Hamilton syringes, at a rate of 0.2µl/min. After the injection, needles were kept in place for 5 more minutes before removal and the scalp incision was sutured. The surgical area was cleaned before and after the procedure using povidone iodine (Vétédine®, Vetoquinol). Respiration rate was monitored and body temperature was maintained at 37±0.5°C with a heating pad during the surgery. Anesthesia was reversed with a subcutaneous injection of atipamezole (0.25 mg/kg; Antisedan®, Vetoquinol). Mice were then placed in a ventilated heating box (25°C) and monitored until full recovery from anesthesia. Postoperative prophylactic pain management consisted of paracetamol administration in drinking water (1.45 ml/20ml of water, Doliprane®, Sanofi) during 48 h.

Behavioral evaluations

The Morris water maze task was used to investigate spatial memory at 4 mpi on $A\beta_{wt}$-, $A\beta_{ice}$-, and PBS-inoculated $APP_{swe}/PS1_{dE9}$ mice. WT littermates injected with PBS were used as controls for the tests. Mice were handled for 2 min per day, during 5 days prior to any test in order to prevent stress bias effects during tasks. An open 122 cm-wide circular swimming arena was maintained at 22°C and exposed to a 400 lux-lighting. This arena was virtually divided into four quadrants. Before the behavioral assessment, all mice took a swimming test. The behavioral assessment was carried out in three phases (habituation, training, probe). At the end of each session, mice were dried with a heated towel before returning to their home cages. During the habituation phase (day 1), mice were trained to find a visible platform to escape from the water. To facilitate its detection, the platform was emerged 0.5 cm above the surface of the water and a colorful cue was placed on it. This phase consisted of four 60-s trials, with an inter-trial interval (ITI) of 20-30 min. For each trial, the starting point was different as well as the location of the platform. When the mice did not find the platform within the 60 s, they were guided to its location and were left on the platform to rest for 30 s. The training phase (day 2 to 5) consisted in three daily 60-seconds trials, with 20-30 min ITI, during four days. For each trial, the starting point was different whereas the location of the platform remained the same. The platform was hidden 0.5 cm beneath the water surface and the cue previously placed on it was removed. Visual cues were placed, from the beginning, around the

maze to serve as spatial landmarks and help mice navigating to the platform. When the mice did not find the platform within the 60 s, again, they were guided to its location and were left on the platform to rest for 30 s. All trials last 60 s or until the animal located the platform. Escape latency, *i.e.* the time required to find the platform, was evaluated during the habituation and the training phases to assess learning abilities. A probe test (day 6) was performed 24 hours after the last training session to assess spatial long-term memory. During this phase, the platform was removed from the maze. Mice were placed in the water for 60 s from a position opposite to the platform. The time spent in each virtual quadrant of the maze was recorded (EthoVision XT 13).

Animal euthanasia and brain preparation

Mice were euthanized at 4 mpi, after the behavioral tests, with an intraperitoneal injection of a lethal dose of pentobarbital (100 mg/kg; Exagon®, Axience). They were perfused intracardiacally with cold sterile PBS (0.1M, pH 7.4) for 4 min, at a rate of 8 ml/min. The brain was extracted and split in two hemispheres. The left hemisphere was dissected in order to take out the hippocampus and the cortex. Samples were directly snap-frozen into liquid nitrogen and stored at −80°C until further biochemical analysis. For histology purpose, the right hemisphere was post-fixed into 4% paraformaldehyde for 48 hours at 4°C, and cryoprotected into a 15% sucrose solution in PBS (0.1M, pH 7.4) for 24 hours and in a 30% sucrose solution for 48 hours at 4°C. Serial coronal sections of 40 µm were performed with a microtome (SM2400, Leica Microsystems) and stored at -20°C in a storing solution (glycerol 30%, ethylene glycol 30%, distilled water 30%, phosphate buffer (1M, pH 7.4, SIGMA 940-8) 10%).

Mouse brain sample preparation for biochemical analyses

For protein extraction, deep-frozen brain samples were dissociated with Collagenase D (2 mg/mL) in Tris-buffered saline (TBS, 20 mM Tris-HCl, 150 mM NaCl, pH= 7.4) at 1:10 (TBS volume:brain wet weight) and incubated at 37°C. Samples were further homogenized using a Dounce homogenizer with 20 strokes in ice-cold quench buffer containing protease inhibitors (cOmplete™, PMSF 1 mM) and phosphatase inhibitors (Na3VO4 1 mM, NaF 10 mM). Sarkosyl (2% in water) was added to homogenates. Samples were centrifuged for 30 min at 10,000 xg at 4°C. The resulting supernatant was further centrifuged for 1 hour at 100000 g at 4°C using a TLA-100.2 fixed angle rotor in a Beckman Optima™ TL 100 Ultracentrifuge. The resulting supernatant called S100K contained the sarkosyl-soluble fraction. Pellet (P100K) was washed twice in TBS and finally resuspended in TBS at 1:10 (TBS volume:brain wet weight). All samples were stored at -80°C until further analysis.

Western blotting and dot blotting

For western blot, samples were diluted in NuPage™ LDS (Lithium Dodecyl Sulfate) sample buffer (ThermoFisher) and NuPAGE™ Sample Reducing Agent (ThermoFisher) so as to load 20 µg of proteins. After heating, samples were loaded on a 4-12% Criterion™ XT Bis-Tris gel (Bio-Rad), migrated in XT MES Running Buffer (Bio-Rad) for 1 hour at 110 V and transferred onto 0.2 µm nitrocellulose. For dot blot, 2µL of samples were directly loaded onto 0.2 µm nitrocellulose (Bio-Rad). After 1 hour of blocking solution (5% skimmed milk in TBS-T, containing TBS 1x, 0.02% Tween20, pH=7.4) at RT, membranes were blotted ON at 4°C with APP-Cter-17 targeting the last 17 amino acids of the human APP sequence (9, 10)(1/2500 in blocking solution, western blot) and A11 targeting oligomeric species (ThermoFisher, 1/2000 in blocking solution, dot blot). After rinsing in TBS-T, membranes were incubated with appropriate horseradish peroxidase (HRP)-conjugated secondary antibodies (Invitrogen, 1/5000 in TBS-T) for 1 hour at RT. Proteins were revealed using ECL™ Western Blotting Detection Reagent (G&E Healthcare). Quantification of protein expression levels were performed with ImageJ (https://imagej.nih.gov/ij/).

Immunohistochemistry and histochemical stains

Aβ deposits were examined using a 4G8 (Biolegend 800706) labeling. Tau was examined using labeling with AT8 (Thermo MN1020B) directed against hyperphosphorylated Tau that binds to a conformational epitope including phosphorylated Thr212 and Ser214. Astrocytes were stained with the GFAP antibody (Dako Z0334). Microglia were stained with Iba1 (Wako 1919741) and CD68 (Biorad MCA1957).

Washing and incubation steps were performed on a shaker at RT unless indicated otherwise. Free-floating brain sections were rinsed in a 0.1M PBS (10% Sigma-Aldrich® phosphate buffer, 0.9% Sigma-Aldrich® NaCl, distilled water) before use. According to the immunolabeling, primary antibody-dependant pretreatment of tissues was performed as follows: 4G8 labeling pretreatment with 70% formic acid (VWR®) for 20 min at RT; AT8 labeling pretreatment with citrate buffer 1X (Diagnostic BioSystems®) for 30 min at 95°C; any pretreatment was needed for GFAP and Iba1 labeling. All tissues were then incubated 20 min in 30% hydrogen peroxide (Sigma-Aldrich®) diluted 1/100 in water, to inhibit endogenous peroxidases.

Blocking of non-specific antigenic sites was achieved over 1 hour using a 0.2% Triton X-100/0.1M PBS (Sigma-Aldrich®) (PBST) solution containing normal goat serum (NGS) at concentrations specific for each labeling (table of reagents). Sections were then incubated at 4°C as follows: with biotinylated 4G8 antibody diluted 1/500 in 3%NGS/PBST for 48 h; with AT8 antibody diluted 1/500 in a 5%NGS/PBST for 96h; Iba1 (Wako 1919741, 1/1000) or with GFAP antibody diluted 1/20000 in a 3%NGS/PBST for 48 h. After rinsing (PBS three times 10 min), except for biotinylated 4G8, an incubation with the appropriate biotinylated secondary antibody, diluted to 1/1000 in PBST, was

performed for 1 hour at RT. Then, a 1 hour incubation at room temperature with a 1:250 dilution of an avidin-biotin-enzyme complex solution (ABC Vectastain® ABC-HRP kit, Vector Laboratories®) and revelation was performed using the DAB Substrate Kit with Nickel, (3,3'-diaminobenzidine) (DAB SK4100 kit, Vector Laboratories®). The AT8 labeling was counter-stained using cresyl-violet (Nissl staining). The GFAP labeling was followed by Periodic Acid Schiff (PAS) staining of polysaccharides according to the manufacturer instructions (PAS-IFU/PAS-2, ScyTek Lab, https://www.scytek.com/). Briefly, slides were immersed in PAS (PAS-IFU/PAS-2, ScyTek Lab) for 5 min. After rinsing, they were immersed again in PAS for 1 min. After a second rinsing, they were counterstained with Hematoxylin and a bluing agent from the supplier, before to be mounted. Sections were mounted on Superfrost Plus slides (Thermo-Scientific®). All sections were dehydrated in successive baths of ethanol at 50°, 70°, 96° and 100° and in xylene. Slides were mounted with the Eukitt® mounting medium (Chem-Lab®) or VectaShield and nail polish for fluorescence.

Co- staining of pTau and Aβ plaques was performed to investigate their colocalization. Free-floating sections were permeabilized in a 0.2% Triton X-100/0.1 M PBS (Sigma-Aldrich®) solution for 3 × 10 min. Free-floating sections were pre-treated with citrate buffer 1X (Diagnostic BioSystems®) for 30 min at 95°C. Sections were blocked in a 10%NGS/PBST solution for 1 h at room temperature before being incubated with the AT8 antibody (Wako 1919741, 1/1000) solution, 4 nights at 4°C. On the next day, sections were rinsed in 0.1 M PBS and incubated for 1 h at room temperature with the appropriate secondary antibody diluted to 1/500 in PBST (anti-rabbit AlexaFluor 633). Sections were then rinsed and stained with 0.02% Thioflavin-S (T1892 Sigma) in 50% ethanol. Sections were mounted on Superfrost Plus (Thermo- Scientific®) slides with the Vectashield ® mounting medium with a refractive index of 1.45. Images of stained sections were acquired using a Leica SP8 confocal optical microscope (TCS SPE) with a 40 × oil-immersion objective (refractive index 1.518) and the Leica Las X software. The excitation wavelengths were 633 nm (for AT8) or 488 nm (for Aβ).

Aβ deposits and Tau-positive neuritic plaques analysis and observation

Stained sections were scanned using an Axio Scan.Z1 (Zeiss® - Z-stack images - 16 planes, 1µm steps with extended depth of focus - acquired at 20× before yielding a projection image). Each section was extracted individually in the .czi format using Zen lite (Zeiss®). Image processing and analysis were performed with ImageJ. Macros were developed for each staining in order to attain a reproducible semi-automated quantification. Images were imported with a 50% reduction in resolution (0.44 µm/pixel), converted to the RGB format and in .tif format. For the 4G8 immunostaining, segmentation was performed through an automatic local threshold using the

Phansalkar method (radius = 15). Aβ load was evaluated after quantification of the 4G8-labeled particles between 8 and 2,000 µm², and normalization to the surface area of each region of interest (ROI). For the AT8 staining, the blue component of each image was extracted to remove the cresyl violet counter-staining from the analysis. AT8-positive tau areas were assessed following an automatic local threshold of the staining with the Phansalkar method (radius=15). In $APP_{swe}/PS1_{dE9}$ mice, tau lesions occur in the form of neuritic plaques *i.e.* tau aggregates within neurites surrounding Aβ deposits. The AT8-positive area presenting within neuritic plaques was evaluated by drawing circular regions of interest (with a constant area of 6µm²), and by quantifying the percentage of tau-positive regions within each ROI, using the thresholding method previously described. All quantifications were performed on adjacent slices between -0.34 mm and -4.36 mm from bregma. Ten adjacent slices were analyzed for the 4G8 and AT8 stainings. All ROIs were manually segmented using the Paxinos and Franklin neuro-anatomical atlas of the mouse brain (11).

Co-stainings of astrocytes and Tau

In order to evaluate Tau lesions within astrocytes, the co-staining of astrocytes and Tau was performed. Free-floating sections were pretreated with citrate buffer 1X (Diagnostic BioSystems®) for 30 min at 95°C following by permeabilization in PBST for 10 min. Blocking of non-specific antigenic sites was achieved over 1 hour using a PBST containing 10% NGS. Slices were incubated with AT8 (Thermo MN1020B, 1/500) and GFAP (Dako Z0334, 1/10000) antibodies diluted in 5% NGS/PBST solution for 96 hours at 4°C. After rinsing (PBST three times 10 min), incubation with secondary antibodies coupled to a fluorochrome (Alexa Fluor 488 and 594) diluted in PBST was then performed for 1 hour at RT. GFAP/AT8 double labeling was counterstained with DAPI. Sections were rinsed and mounted on Superfrost Plus slides (Thermo-Scientific®) with the Vectashield® mounting medium (refractive index of 1.45). Images of stained sections were acquired using a DMI6000 confocal optical microscope (TCS SPE, Leica) with a 40x oil-immersion objective (refractive index of 1.518) and the Leica Las X software. A confocal zoom of 3 and a pinhole aperture fixed at 1 Airy were applied. Acquisition was performed in sequential mode with a sampling rate of 1024 × 1024 and a scanning speed of 700 Hz. Image resolution was 60 nm/pixel and the optical section was 0.896 µm. Twelve separate planes with a 0.1 µm step were acquired. The excitation wavelengths were 488 nm (for Tau) or 594 nm (for astrocytes).

Evaluation of synaptic density

Synaptic density was assessed in the hippocampus (CA1) and the perirhinal/entorhinal cortex of all inoculated mice using a double immunolabeling of presynaptic (Bassoon) and postsynaptic

(Homer1) markers. Free-floating sections were permeabilized in a 0.5% Triton X-100/0.1M PBS (Sigma-Aldrich®) solution for 15min. Slices were incubated with Bassoon (Abcam Ab82958, 1/200) and Homer1 (Synaptic systems 160003, 1/400) antibodies diluted in 3% BSA/PBST solution for 24 hours at +4°C. Incubation with secondary antibodies coupled to a fluorochrome (Alexa Fluor 594 and 633) diluted in a 3% BSA/0.1M PBS solution was then performed for 1h at room temperature. Sections were rinsed and mounted on Superfrost Plus slides (Thermo-Scientific®) with the Vectashield® mounting medium (refractive index of 1.45). Images of stained sections were acquired using a DMI6000 confocal optical microscope (TCS SPE, Leica) with a 63x oil-immersion objective (refractive index of 1.518) and the Leica Las X software. A confocal zoom of 3 and a pinhole aperture fixed at 1 Airy were applied. Acquisition was performed in sequential mode with a sampling rate of 1024 x 1024 and a scanning speed of 700 Hz. Image resolution was 60 nm/pixel and the optical section was 0.896 µm. Twenty six separate planes with a 0.2 µm step were acquired. The excitation wavelengths were 594 nm or 633 nm. Image acquisition in the CA1 region was performed on 4 adjacent slices located between -1.82 mm and -3.28 mm from the bregma, with 2 images per slice. For the perirhinal/entorhinal cortex, 3 adjacent slices located between -3.28 mm and -4.24 mm from the bregma were analyzed, with 2 images acquired per slice. A 3D deconvolution of the images was performed using the AutoQuant X3 software. The deconvoluted 8-bit images were analyzed using the ImageJ software. Briefly, automated 3D segmentation of the presynaptic (Bassoon) and postsynaptic (Homer1) stained deconvoluted images was performed using "3D spots segmentation" from ImageJ (with "gaussian fit", "block" and "no watershed" options; https://imagej.net/plugins/3d-segmentation). Co-localization of overlapping objects was evaluated using "DiAna" from imageJ (https://imagej.net/plugins/distance-analysis). The percentage of colocalized objects was quantified as an index of synaptic density.

Colocalisation between microglia lysosomes and Homer synaptic marker

In order to evaluate microglial engulfment of synapse, the co-staining of microglia (anti-CD68) and postsynaptic (Homer1) markers were performed. Free-floating sections were permeabilized in a 0.3% Triton X-100/0.1M PBS (Sigma-Aldrich®) solution for 30 min. Slices were then rinced in a 0.02% Tween/0.1M PBS (Sigma-Aldrich®) solution for 5 min following by further permeabilization in 0.3% Triton X-100/0.1M PBS (Sigma-Aldrich®) solution for 3X5min. Sections were blocked in a 3% NGS, 3%BSA, 0.3% Triton X-100/0.1M PBS (Sigma-Aldrich®) solution for 1h at RT before being incubated with the CD68 antibody (MCA1957, Biorad, 1/500) overnight at 4°C. On the next day, sections were rinsed in 0.1M PBS and incubated for 2h at RT with the appropriate secondary antibody diluted to 1/1000 in PBS (anti-rat AlexaFluor 488). Sections were rinsed in 0.1M PBS and permeabilized in a 0.5% Triton X-100/0.1M PBS (Sigma-Aldrich®) solution for 15 min. Slices were incubated with

Homer1 (Synaptic systems 160003, 1/400) antibodies diluted in 3% BSA/PBST solution for 24 hours at RT. Incubation with appropriate secondary antibodies coupled to a fluorochrome (Alexa Fluor) diluted in a 3% BSA/0.1M PBS solution was then performed for 1h at room temperature. Sections were rinsed and mounted on Superfrost Plus (Thermo-Scientific®) slides with the Vectashield® mounting medium with a refractive index of 1.45. Images of stained sections were acquired using a Leica DMI6000 confocal optical microscope (TCS SPE) with a 63x oil-immersion objective (refractive index 1.518) and the Leica Las X software. A confocal zoom of 1.28 and a pinhole aperture fixed at 1 Airy were applied. Acquisition was performed in sequential mode with a sampling rate of 1024 x 1024 and a scanning speed of 700 Hz. Image resolution was 80 nm/pixel and the optical section was 0.896 µm. Seventy three separate planes with a 0.2 µm step were acquired. The excitation wavelengths were 488 nm (microglia) or 633 nm (Homer). Image acquisition in the hippocampus was performed on 2 adjacent slices located between -1.58 mm and -3.40 mm from bregma, with 4 images per slice. 3D reconstruction of microglia and synaptic spots was performed with Imaris (9.7). First, confocal images were converted in Imaris supported file by Imaris File Converter (9.7). While mean filter gradients were applied to CD68 images, subtraction background was performed on synaptic images. Then, the plug-in "surface" was used to reconstruct the CD68 volume and the plug-in "spot" to reconstruct Homer volume. Finally, the XTension file "Split spots into surface objects" (Matlab) was used to calculate the number of synaptic spots in CD68 positive surface.

Western bot analysis of phosphorylated Tau in hippocampal lysates

The cortex and the hippocampal tissues were removed and homogenized in a RIPA lysis buffer accommodating (50 mM Tris-HCl, pH 7.4; 150 mM NaCl; 1% Triton X-100; 1% sodiumdeoxycholate; 0.1% SDS; 1 mM EDTA) containing a cocktail of protease and phosphatase inhibitors 1% (v/v). Then lysates were sonicated 2x 1sec 20% amp and dosed for proteins using the BCA method. Samples in reduced Laemli loading buffer were boiled for 5 min, and equal amounts of protein (20 µg) were resolved on 10% stain free Bis-Tris polyacrylamide precast gels (Bio-Rad) in denaturing conditions. Proteins were transferred to a polyvinylidene difluoride 0.2 mm membranes (Millipore). Membranes were blocked with Tris-buffered saline (10 mM Tris and 150 mM NaCl, pH 7.4) containing 0.01% Tween 20 and 5% bovine serum albumin (grade V, Sigma) for 1 h at room temperature. Membranes were then incubated overnight at 4°C with the following primary antibodies: pan Tau (1:1000 dilution; catalog #A0024, DAKO); Phospho-Tau (Ser202, Thr205) (AT8; 1:1000 dilution; catalog # MN1020, Invitrogen). After cleaning with Tris- buffered saline with Tween 20 (TBS-T) (0.05%), membranes were incubated with the appropriate horseradish peroxidase-conjugated secondary antibodies (1:40,000; Jackson ImmunoResearch and Immunotech) for 45 min

at room temperature. Specific proteins were visualized with an enhanced chemiluminescence ECL Detection System (Bio-Rad). Chemiluminescence detection was performed with the Bio-Rad Chemidoc system and analyzed with the ImageJ software.

Statistical analysis

Statistical analysis was performed using the GraphPad Prism software 9. Data normality and variance homogeneity were evaluated using Shapiro-Wilk and Cochran C tests, respectively. When the data do not follow the normal distribution or the sample size was too small, we applied nonparametric tests such as Kruskal-Wallis (alternatively to ANOVA) and Mann-Whitney U (alternatively to t-test) test. We used two-sided tests. For spine density comparisons in neurons, Kruskal-Wallis with Dunn's multiple comparisons were performed. For the behavioral tasks analysis, Kruskal-Wallis tests with Dunn's multiple comparisons were performed except when repeated measures were acquired. In this case, a two-way repeated measures ANOVA with the Geisser-Greenhouse correction and Dunnett's multiple comparisons was carried out. For the post-mortem analysis, Kruskal-Wallis tests with Dunn's multiple comparisons tests were performed in order to compare differences between inoculated mice. The significance level was set at $p<0.05$. Data are shown on scattered dot plots with mean ± standard error of the mean (s.e.m).

Data availability

The data that support the findings of this study are available from the corresponding author, upon request.

Declarations

Ethics approval and consent to participate

All experimental procedures were conducted in accordance with the European Community Council Directive 2010/63/UE and approved by local ethics committees (CEtEA-CEA DSV IdF N°44, France) and the French Ministry of Education and Research (APAFIS#21333-2019062611099838 v2 authorization given after depositing the research protocol and associated ethic issues), and in compliance with the 3R guidelines. Animal care was supervised by a dedicated veterinarian and animal technicians.

Availability of data and materials

The data that support the findings of this study are available from the corresponding author.

Author's contributions

M.C., A.S.H., A.B., M.D. contributed to the study conception and design. M.J.S., E. B., A.B. provided the recombinant Aβ proteins. M.C. performed the inoculations in mice. M.C. designed and

performed memory evaluations, M.C., A.S.H., F.P. designed and performed the immunohistological analysis in animals. M.C., M.J.S., A.B. performed biochemical analysis. M.J.S., E. B., A.B. performed *ex vivo* experiments on cell cultures. FB performed electrophysiology on mouse splice. M.C., A.B., M.D. wrote the manuscript. All authors commented on previous versions of the manuscript. All authors read and approved the final manuscript.

List of abbreviations

Aβ = amyloid-β; APP = amyloid precursor protein; AD = Alzheimer's disease; ICE = Icelandic; LTP = long-term potentiation; mpi = months post-inoculation; WT = wild-type.

REFERENCES

1. Leveille F, El Gaamouch F, Gouix E, Lecocq M, Lobner D, Nicole O, et al. Neuronal viability is controlled by a functional relation between synaptic and extrasynaptic NMDA receptors. Faseb J. 2008;22(12):4258-71.
2. Riedl J, Crevenna AH, Kessenbrock K, Yu JH, Neukirchen D, Bista M, et al. Lifeact: a versatile marker to visualize F-actin. Nat Methods. 2008;5(7):605-7.
3. Rodriguez A, Ehlenberger DB, Dickstein DL, Hof PR, Wearne SL. Automated three-dimensional detection and shape classification of dendritic spines from fluorescence microscopy images. PLoS ONE. 2008;3(4):ARTN e1997.
4. Celestine M, Jacquier-Sarlin M, Borel E, Petit F, Perot JB, Herard AS, et al. Long term worsening of amyloid pathology, cerebral function, and cognition after a single inoculation of beta-amyloid seeds with Osaka mutation. Acta Neuropathol Commun. 2023;11(1).
5. Garcia-Alloza M, Robbins EM, Zhang-Nunes SX, Purcell SM, Betensky RA, Raju S, et al. Characterization of amyloid deposition in the APPswe/PS1dE9 mouse model of Alzheimer disease. Neurobiol Dis. 2006;24(3):516-24.
6. Jankowsky JL, Fadale DJ, Anderson J, Xu GM, Gonzales V, Jenkins NA, et al. Mutant presenilins specifically elevate the levels of the 42 residue beta-amyloid peptide in vivo: evidence for augmentation of a 42-specific gamma secretase. Hum Mol Genet. 2004;13(2):159-70.

7. du Sert NP, Hurst V, Ahluwalia A, Alam S, Avey MT, Baker M, et al. The ARRIVE guidelines 2.0: Updated guidelines for reporting animal research. Bmc Vet Res. 2020;16(1):Article 242.

8. Gary C, Lam S, Herard AS, Koch JE, Petit F, Gipchtein P, et al. Encephalopathy induced by Alzheimer brain inoculation in a non-human primate. Acta Neuropathol Commun. 2019;7(1):126.

9. Sergeant N, David JP, Champain D, Ghestem A, Wattez A, Delacourte A. Progressive decrease of amyloid precursor protein carboxy terminal fragments (APP-CTFs), associated with tau pathology stages, in Alzheimer's disease. J Neurochem. 2002;81(4):663-72.

10. Vingtdeux V, Hamdane M, Gompel M, Begard S, Drobecq H, Ghestem A, et al. Phosphorylation of amyloid precursor carboxy-terminal fragments enhances their processing by a gamma-secretase-dependent mechanism. Neurobiol Dis. 2005;20(2):625-37.

11. Paxinos G, Franklin KBJ. The mouse brain in stereotaxic coordinates. second ed. Press A, editor. San Diego: Academic Press; 2001.
