# Supplementary material for: Transmissible long-term neuroprotective and pro-cognitive effects of 1–42 beta-amyloid with A2T icelandic mutation in an Alzheimer’s disease mouse model
Source: Mol Psychiatry. 2024 Jun 14;29(12):3707–21. doi: 10.1038/s41380-024-02611-8 (PMC11609088; doi:10.1038/s41380-024-02611-8)
Supplement: Supplementary file 3 — Supplementary Table 1 [file 41380_2024_2611_MOESM3_ESM.pdf]

SUPPLEMENTARY TABLE 1

Transmissible long-term neuroprotective and pro-cognitive effects of 1-42 beta-amyloid with A2T icelandic mutation in an Alzheimer's disease mouse model

Marina Célestine[1,2], Muriel Jacquier-Sarlin[3], Eve Borel[3], Fanny Petit[1,2], Fabien Lante[3], Luc Bousset[1,2], Anne-Sophie Hérard[1,2], Alain Buisson[3], Marc Dhenain[1,2]

Author affiliations:

[1] Université Paris-Saclay, CEA, CNRS, Laboratoire des Maladies Neurodégénératives, 18 Route du Panorama, F-92265 Fontenay-aux-Roses, France.

[2] Commissariat à l'Energie Atomique et aux Energies Alternatives (CEA), Direction de la Recherche Fondamentale (DRF), Institut de Biologie François Jacob, MIRCen, 18 Route du Panorama, F-92265 Fontenay-aux-Roses, France

[3] Univ. Grenoble Alpes, Inserm, U1216, Grenoble Institut Neurosciences, GIN, 38000 Grenoble, France

Correspondence to: Marc Dhenain, DVM, PhD

Université Paris-Saclay, CEA, CNRS, Laboratoire des Maladies Neurodégénératives, MIRCen, 18 Route du Panorama, F-92265 Fontenay-aux-Roses, France

E-mail: Marc.Dhenain@cnrs.fr

Key resource table

Reagent or Resource	Source	Identifier
**Antibodies**		
Goat anti-mouse secondary antibody Alexa Fluor™ 633nm Dilution: 1/500	Invitrogen	Cat#A21050
Anti-mouse biotinylated secondary antibody Dilution: 1/1000	Vector Laboratories	Cat#BA9200
Goat anti-rabbit secondary antibody Alexa Fluor™ 594nm Dilution: 1/500	Alexa Fluor	Cat#A11012
Donkey anti-Rabbit IgG Secondary Antibody, HRP Dilution: 1/5000	Invitrogen	Cat#A16023
Mouse anti-Bassoon monoclonal antibody Dilution: 1/200	Abcam	Cat#Ab82958
Rabbit anti-Iba1 monoclonal antibody Dilution: 1/1000	Wako	Cat#19_19741
Mouse anti-CD68 monoclonal antibody Dilution: 1/500	Biorad	Cat#MCA1957
Mouse anti-phospho-Tau (Ser202, Thr205)	Thermo	Cat#MN1020B

monoclonal antibody (AT8) Dilution: 1/500		
Mouse Anti-β-Actin monoclonal antibody Dilution: 1/5000	Sigma-Aldrich®	Cat#A1978
Mouse anti-β-amyloid monoclonal antibody (4G8), biotinylated Dilution: 1/500	Biolegend	Cat#800706
Mouse Anti-γ-Tubulin monoclona antibody Dilution: 1/5000	Sigma-Aldrich®	Cat#T6557
Rabbit anti-APP-Cter-17 antibody Dilution: 1/2500	Gift from [Sergeant, 2002; Vingtdeux, 2005]	
Rabbit anti-Glial fibrillary acidic protein (GFAP) polyclonal antibody Dilution: 1/10000	Dako	Cat#Z0334
Rabbit anti-Homer1 monoclonal antibody Dilution: 1/400	Synaptic systems	Cat#160003
Rabbit oligomer A11 Polyclonal antibody Dilution: 1/2000	ThermoFisher	Cat#AHB0052
Rabbit recombinant anti-Amyloid Precursor Protein monoclonal antibody (Y188) Dilution: 1/2000	Abcam	Cat#Ab32136
**Chemicals and commercial assay or kit**		
ABC Vectastain® ABC-HRP kit	Vector Laboratories®	Cat#PK6100
Atipamezole	Antisedan®, Vetoquinol	
Benzonase	This paper	
Bovine serum albumin (BSA)	Sigma-Aldrich®	Cat#A7906
Citrate Buffer 10X, pH 6.0	Diagnostic BioSystems®	Cat#924
Collagenase D	Sigma-Merck	Cat#11088866001
cOmplete™ protease inhibitor cocktail	Roche	Cat#4693116001
Cresyl violet	Merck	Cat#10510-54-0
Culture medium	See composition in this paper	
DAB Substrate kit, Peroxidase (HRP), with Nickel	Vector Laboratories®	Cat#SK4100

DMEM, high glucose, GlutaMAX™ Supplement	ThermoFisher	Cat#10566016
Dulbecco's Modified Eagle's Medium (DMEM)	Sigma-Aldrich®	Cat#D6671
Dulbecco's phosphate saline (DPBS) 1X	Gibco™, ThermoFisher	Cat#14190094
ECL™ Western Blotting Detection Reagent	GE Healthcare	Cat#28980926
Ethanol absolute	VWR	Cat#83813360
Ethylene glycol	Carlo Erba	Cat#346502
Eukitt® mounting medium	Chem-Lab®	Cat#50980467
Fluorsave	VWR	Cat#345789-20
Foetal bovine serum	Sigma-Aldrich®	Cat#F7524
Formic acid	VWR®	Cat#BDH4554
Glutamine	Thermofisher	Cat#A2916801
Glycerol	Fisher	Cat#12144481
Horse serum	Gibco™, ThermoFisher	Cat#16050122
Hydrogen peroxide 30%	Sigma-Aldrich®	Cat#H1009
Imidazole	Sigma-Aldrich®	Cat#56750
In-Fusion HD Cloning kit	Takara	Cat#638943
Isopropyl-beta-D-thiogalactopyranoside (IPTG)	Euromedex	Cat#EU0008-B
Ketamine	Imalgène® 1000, Merial	
Kinetic Limulus amebocyte lysate (LAL) chromogenic endotoxin quantitation kit	Pierce™ Thermo Scientific	Cat#PIER88282
Laminin	Sigma-Aldrich®	Cat#L2020
Lidocaine	0.5% Xylovet®, Ceva Santé Animale	
Liquid nitrogen	Air products, France	
Luria broth (LB medium)	Euromedex	Cat#AE0103
$MgCl_2$	Merck	Cat#M8266-100G
Na3VO4	Merck	Cat#450243-10G
NaF	Merck	Cat#S6776-100G
Nickel-nitriloacetic acid (Ni-NTA) column	Macherey nagel	Cat#7454105
Normal goat serum (NGS)	Sigma	Cat#G6767
NuPAGETM Lithium Dodecyl Sulfate (LDS) sample buffer 4X	ThermoFisher	Cat#NP0007
NuPAGE™ Sample Reducing Agent 10X	ThermoFisher	Cat#NP0004

Paracetamol	Doliprane®, Sanofi	
Paraformaldehyde, PFA	Sigma	Cat#P7148
Penicillin/Streptomycin	Invitrogen	Cat#15140-122
Pentobarbital	Exagon®, Axience	
Phosphate buffer solution, 1 M , pH 7.4	Sigma-Aldrich®	Cat#P3619
Phosphate Buffered Saline (PBS), pH 7.4	Sigma-Aldrich®	Cat#806552
PMSF, 0.1M in ethanol	Merck	Cat#93482
Poly-D-lysine hydrobromide	Sigma-Aldrich®	Cat#P6407
Povidone iodine	Vétédine®, Vetoquinol	
Sarkosyl	Merck	L5777-100G
Sodium chloride (NaCl)	Sigma-Aldrich®	Cat#S9888
Sucrose	Sigma-Aldrich®	Cat#S0389
Thioflavine T	Wako Chemical Industries Ltd, Osaka, Japan	
Tris-HCl	Merck	Cat#252859-500G
Triton X-100	Sigma-Aldrich®	Cat#X100
Ultra TMB-ELISA	Pierce™1-Step™ , Thermo Scientific	Cat#34021
Urea	Euromedex	Cat#EU0014B
Xylazine	2% Rompun®, Bayer Healthcare	
Xylene	VWR Chemicals	Cat#28973363
**Equipements**		
60x water-immersion objective	Nikon, melville, ny, usa	
40x water-immersion objective	Leica	
Argon laser	Nikon, melville, ny, usa	
Axio Scan.Z1	Zeiss®	
Centrifuge	Multifuge X1R Heraeus Thermoscientific	
Confocal optical microscope (TCS SPE)	Leica DMI6000	
EPC 10 Amplifier Patchmaster Multi-channel	HEKA Elektronik Dr. Schulze gmbh, Wiesenstrasse, Germany	
FACS	Becton Dickinson FACScanto II	
Heating box	Phymep	
Heating pad	Phymep	
Microtome	Leica Vt1200 blade	
Open circular swimming arena	This paper	
OptimaTM TL 100 Ultracentrifuge	Beckman	
Perfusion pump	Fisher Scientific	Cat#1170-5369
SM2400 microtome	Leica Microsystems	
Sonicator	Fischerbrand	
Spark plate reader	Tecan	

Spectrophotometer	Eppendorf biophotometer plus	
Stereotaxic frame	Phymep, France	
SW32ti swinging bucket rotor	Beckman	
Ti C2 confocal microscope	Nikon, melville, ny, usa	
TLA-100.2 fixed angle rotor	Beckman	
DMI6000 confocal optical microscope	TCS SPE, Leica	
**Experimental models: cell lines + bacteria**		
Escherichia coli bl21 (dE3)	New england BioLabs (C2987H)	
HEK293T cells	ATCC (CRL-3216)	
**Experimental models: Organisms / Strains**		
APPswe/PS1dE9, C57Bl6 background, male	Jackson Laboratories	JAKS-5864
OF1/SWISS	Janvier	
WT C57Bl6	Jackson Laboratories	JACKSIMA-0664
**Gel, membranes**		
Bis-Tris gel 4-12% Criterion™ XT	Bio-Rad	Cat#3450125
Nitrocellulose membrane, 0.2 µm	Bio-Rad	Cat#1620112
Precision Plus Protein™ Dual Xtra Prestained Protein Standards	Bio-Rad	Cat#1610377
SDS-PAGE gel	This paper	
XT MES Running Buffer	Bio-Rad	Cat#1610789
**Genetic reagent: Plasmids, cDNA**		
cDNAs of human APP695	Dr Rémi Sadoul (Grenoble Institute of Neuroscience)	
cDNAs of Iceland mutant	This paper	
cDNAs of Swedish mutant	This paper	
Life-actin-GFP	Addgene	
pCMV-VSV-G plasmid	Addgene	
pet28a-vector	Novagen, Paris, France	
pLenti-C-mCherry vector	Dr Christophe Bosc (Grenoble Institute of Neuroscience)	
pmcherry-N1 vector	Snapgene	

psPAX2 plasmid	Addgene	
Synthetic oligonucleotides	Sigma, Lyon, France	
**Material**		
24-well plates	Falcon; Beckton Dickinson	
35 mm glass-bottom dishes	MatTek	
Coverslips	MatTek	
Stimulating bipolar microelectrodes	World Precision Instruments, Inc.	
Recording glass pipette filled with ACSF	Harvard Apparatus	
sterile 96-well plate	BD Falcon	Cat#353916
34-gauge needles	Centravet, Dinan,France	
Hamilton syringes	Centravet, Dinan,France	
Superfrost Plus slides	Thermo-Scientific®	
**Peptides**		
Fusion protein Aβ(His) with wild-type human β-amyloid 1-42 protein (Aβwt)	this paper	
Fusion protein Aβ(His) with mutant human β-amyloid 1-42 protein bearing the A2T mutation (Aβice)	this paper	
Synthetic Aβ1-42	Covalb, France	
**Software and algorithms**		
Patchmaster Multi-channel data acquisition software	HEKA Elektronik Dr. Schulze GmbH, Wiesenstrasse, Germany	
NIS-Elements software	Nikon, Melville, NY, USA	
AutoQuantX3 software	Media Cybernetics, Abingdon, Oxon, UK	
NeuronStudio software package	https://biii.eu/neuronstudio, CNIC – Mount Sinai School of Medicine	
Ethovision XT 13	Noldus https://www.noldus.com/ethovision-xt	
ImageJ Software	https://imagej.nih.gov/ij/download.html	
Zen 2.0 software	Zeiss®, https://www.zeiss.com/microscopy/fr/produits/logiciel/zeiss-zen-lite.html	
Las X software	Leica	
AutoQuant X3 software	https://www.meyerinst.com/mediacybernetics/autoquant/	

ImageJ - 3D spots segmentation	https://imagej.net/plugins/3d-segmentation
ImageJ - DiAna	https://imagej.net/plugins/distance-analysis
Imaris 9.7	https://imaris.oxinst.com/
GraphPad Prism software 9	https://www.graphpad.com/
